# Supplementary material for: Shaping care home COVID-19 testing policy: a protocol for a pragmatic cluster randomised controlled trial of asymptomatic testing compared with standard care in care home staff (VIVALDI-CT)
Source: BMJ Open. 2023 Nov 14;13(11):e076210. doi: 10.1136/bmjopen-2023-076210 (PMC10649600; doi:10.1136/bmjopen-2023-076210)
Supplement: Supplementary data [file bmjopen-2023-076210supp001.pdf]

Supplementary File 1: Description of work packages in the VIVALDI-CT

Work Package	Aims	Participants	Methods	Data collection	Data analysis	Outcomes
WP1	To coproduce a sustainable testing intervention for care home staff.	Care home staff, providers and policymakers.	A series of workshops with care home stakeholders (e.g., home managers, staff, providers) and policymakers (including those with knowledge of testing logistics).<br><br>A workshop will also take place with residents with capacity to consent, relatives and visitors.	1) Consolidation of existing insights into routine testing gleaned through past experiences with the COVID-19 pandemic; 2) discussion of the intervention prototype; and 3) Operationalisation of intervention in ways which are likely to be acceptable and appropriate within the sector.	Data (transcript, detailed notes, images) will be analysed thematically to further develop the initial programme theory specifying important elements of the context, key intervention components, their mechanisms, and their relation to a hierarchy of outcome measures (e.g., health, social and financial outcomes).	Development of a testing intervention for use in WP2
WP2	To evaluate the effectiveness of the testing intervention compared to the recommended testing protocol that is in place at the time in a pragmatic cluster randomised trial.	Care home staff.	A cluster randomised controlled trial in which asymptomatic care home staff will be randomised at provider level to either test twice weekly using LFDs or to follow current national testing guidance.	At enrolment, providers will complete a checklist summarising characteristics of each participating care home. Each week during the testing period care home managers will be asked to record: 1) dates of hospital admissions for residents and their corresponding NHS	Analysis of the primary outcome, and secondary outcomes expressed as event incidence, will be based on Poisson or negative binomial regression with cluster-robust standard errors, adjusting for calendar time and key care home characteristics used in the restricted	The primary outcome will be the incidence of COVID-19 related hospital residents.

				numbers and/or identifiers, 2) number of staff and residents in each care home. The study will collect limited individual-level identifiable data from residents to ensure that the primary outcome of hospital admissions is accurate. Linkage to other routine datasets (hospitalisations, cause of death, COVID-19 test results, vaccination status).	randomisation such as provider, region, and size.	
WP3A	To understand the intervention roll out and identify areas for optimisation to inform future intervention scale-up, should the testing approach prove effective and cost effective	Care home staff	The intervention programme theory and associated logic models from WP1 will be used to undertake a parallel mixed methods process evaluation.<br>Interviews and focus groups will be undertaken with staff.	Data collection will be facilitated though virtual platforms (according to participant norms).<br><br>Quantitative data will include descriptive statistics from across all trial sites complemented by multivariate analyses of data sets where appropriate.	A combination of deductive and inductive thematic analysis will be used.<br><br>Interview/focus group transcriptions and qualitative survey data will be imported into NVivo10 software to facilitate data handling, organisation and coding. Analysis will firstly be	Implementation guidance and training packages ready for future scale up;<br><br>Details of minimal care home requirements and staff competencies necessary for

				Qualitative data will be collected from 28 (10%) care homes evenly distributed across each intervention and control arms and spaced across time. Selection criteria will focus on geography, socio-economic status of area, highest/lowest rates of infection. Heterogeneous samples of staff will be recruited. After consent is attained qualitative data will be collected primarily using focus groups. One to one interviews will also be possible if requested.	thematic and use a combination of deductive and inductive thematic analysis.	intervention delivery.
WP3B	To conduct a mixed methods, process evaluation and exploratory analysis to explore the impact of testing and outbreaks on social care related quality of life in home care residents	Care home residents	The Adult Social Care Outcomes Toolkit (ASCOT) will be used to assess the impact of the intervention and outbreaks on residents. ASCOT is a well-established tool for measuring social care related quality of life	In person interviews. If in-person resident interviews are not possible, Essential Caregivers (designated visitor allowed even during outbreaks) or members of staff will be interviewed.	The ASCOT tool will be used to assess SCRQoL at baseline, during/immediately after an outbreak or mid study, and at end of the study. ASCOT data will be combined with individual level demographic and health and aggregate	Outputs will also be integrated with findings from WP3A to provide a holistic assessment of the acceptability and feasibility of the testing intervention.

			(SCRQoL). We will establish an ASCOT cohort of six care homes stratified by type and size, distributed equally across control and intervention arms. Homes taking part in the qualitative interviews in WP3A are excluded.		home level data to give a comprehensive description of SCRQoL.	
WP4A	To evaluate the costs and benefits of the testing intervention.	NHS, providers, residents, families and staff.	We will examine within-trial costs and outcomes in intervention and control groups from each perspective. We will examine the cost-effectiveness of the intervention in terms of the primary outcome and in terms of all-cause mortality. The costs of admission will be excluded from the total costs under consideration in this case. We will also examine cost-effectiveness in terms of the secondary outcomes	We will examine the cost-effectiveness of the intervention in terms of the primary outcome, excluding cost of admission from total cost. We will also examine cost-effectiveness in terms of secondary outcomes of cases prevented and resident deaths prevented. We will examine outcomes of hospital admission and number of outbreaks alongside costs offset/additional costs	Generalised linear models appropriate to counts (hospital admissions, numbers of outbreaks, cases) or binary outcomes (deaths) and costs will be applied. For the purposes of the cost-effectiveness analyses, these will take into account possible correlations between costs and outcomes either by non-parametric bootstrapping of separate regressions or joint modelling approaches such as seemingly unrelated	Cost-effectiveness of the intervention in terms of the primary trial outcome (incidence of COVID-19 related hospital admission in residents.)<br><br>Cost-effectiveness in terms of the secondary outcomes of cases prevented and resident

			of cases prevented and resident deaths prevented. We will examine the outcomes of hospital admission and number of outbreaks alongside costs offset/additional costs incurred in a cost-consequences analysis.	incurred in a cost-consequences analysis. investigate the cost-effectiveness and cost-consequences of the testing intervention taking both a NHS, Personal Social Services, and a societal perspective.	regressions. Where individual level data are available, analyses will take a multilevel approach to adjust for clustering at the care home level either by two-stage bootstrapping of separate regressions or simultaneous modelling. Incremental cost-effectiveness ratios will be presented, net benefit calculated over a range of willingness to pay values for gains in outcomes to generate cost-effectiveness acceptability curves.	deaths prevented.<br><br>Cost-consequences of hospital admission and number of outbreaks alongside costs offset/additional costs incurred.
WP4B	To model costs and benefits of the testing intervention under different scenarios	NHS, providers, residents, families and staff.	A compartmental model will be built to study transmission of infection and infer the proportion of COVID-19 infections and deaths averted by the intervention under different epidemiological	Data from WP4A will be used.	The model will consider two populations i.e., home residents and staff, and take into account both symptomatic and asymptomatic cases, as well as hospital admissions and deaths.	Estimate of the projected cost-effectiveness of the intervention.

			scenarios (e.g., high/low community incidence of infection, care home population size etc).<br><br>Two scenarios will be modelled: 1) standard care: residents and staff get tested only if they show symptoms; and 2) intervention: standard care plus regular testing of all staff for COVID-19.		The model will be calibrated to trial results and modelling results will be projected in time by extending the time horizon. Unit costs calculated in the first part of the economic analysis will be discounted to future years values and associated to modelling results to estimate the projected cost-effectiveness of the intervention.<br><br>We will also explore the short and long-term costs of the testing intervention under different epidemiological scenarios (e.g., high/low community incidence of infection).	
WP5	To coproduce recommendations on the use of	Providers, care home staff, the NHS and	Three round-table discussions for a	Discussions will be based on world café methodological	Data (transcript, detailed notes, images) will be analysed thematically.	Formal mechanism to ensure

	regular testing for policymakers	primary care, policymakers and public health teams at national and regional / local level, community, residents and families.	maximum of 20 stakeholders.	principles. The views of the community, residents and families will be represented through organisations such as Healthwatch, the Residents and Relatives association and the VIVALDI-CT PPI group.		stakeholders are aware and involved in the work as it progresses and to enable the sector to prepare and plan for results and their implications.<br><br>Production of recommendations on the use of regular testing for policymakers.
