# Supplementary material for: Shaping care home COVID-19 testing policy: a protocol for a pragmatic cluster randomised controlled trial of asymptomatic testing compared with standard care in care home staff (VIVALDI-CT)
Source: BMJ Open. 2023 Nov 14;13(11):e076210. doi: 10.1136/bmjopen-2023-076210 (PMC10649600; doi:10.1136/bmjopen-2023-076210)
Supplement: Supplementary data [file bmjopen-2023-076210supp002.pdf]

Supplementary File 2: Participant Timeline

**Trial visit number**	**Baseline**	**Care home data collection period**							
**Month**	**Month 0**	**Months 1-4**		**Survey**	**Routine COVID-19 datastore**	**Uploaded to Foundry by provider data manager**	**To be reported as and when occurs**	**Weekly collection from providers**	**Participants**
		**Sites on Intervention arm**	**Sites on Standard Care arm**						
Demography	x			x	x				
Resident registry (patient type)	x			x					
Vaccination status	x				x				
Care home characteristics	x			x	x				
Number of residents per home (weekly)		x	x					x	
COVID-associated hospital admission events in residents		x	x		x	x			
Number of staff absent from work		x	x					x	
Total shifts at home this week (sum for all staff)		x	x					x	
Number of agency shifts		X	x					x	

Number of staff per home (weekly)		x	x					x	
Number of staff opting out of asymptomatic testing (weekly)*		x						x	
Routine data on LFD and PCR tests (staff+residents)		x	x		x				
Routine data on hospital admissions (residents)		x	x		x				
Routine data on mortality (residents)		x	x		x				
Outbreak event data		x	x					x	
Safety reporting		x	x				x	x	
Process Evaluation (3a)									
Focus groups		x	x						x
Interviews		x	x						x

* If the control arm starts routine testing, then collected from both arms
