# Supplementary material for: Shaping care home COVID-19 testing policy: a protocol for a pragmatic cluster randomised controlled trial of asymptomatic testing compared with standard care in care home staff (VIVALDI-CT)
Source: BMJ Open. 2023 Nov 14;13(11):e076210. doi: 10.1136/bmjopen-2023-076210 (PMC10649600; doi:10.1136/bmjopen-2023-076210)
Supplement: Author's manuscript [file bmjopen-2023-076210.draft_revisions.pdf]

BMJ Open is committed to open peer review. As part of this commitment we make the peer review history of every article we publish publicly available.

When an article is published we post the peer reviewers' comments and the authors' responses online. We also post the versions of the paper that were used during peer review. These are the versions that the peer review comments apply to.

The versions of the paper that follow are the versions that were submitted during the peer review process. They are not the versions of record or the final published versions. They should not be cited or distributed as the published version of this manuscript.

BMJ Open is an open access journal and the full, final, typeset and author-corrected version of record of the manuscript is available on our site with no access controls, subscription charges or pay-per-view fees (http://bmjopen.bmj.com).

If you have any questions on BMJ Open's open peer review process please email info.bmjopen@bmj.com

Shaping care home COVID-19 testing policy: A pragmatic cluster randomised controlled trial of asymptomatic testing compared to standard care in care home staff (VIVALDI-CT).

Journal:	*BMJ Open*
Manuscript ID	bmjopen-2023-076210
Article Type:	Protocol
Date Submitted by the Author:	31-May-2023
Complete List of Authors:	Adams, Natalie; Royal Free London NHS Foundation Trust, ; UCL Institute of Health Informatics,<br>Stirrup , Oliver ; University College London, Institute for Global Health<br>Blackstone, James; University College London, Comprehensive Clinical Trials Unit<br>Krutikov, Maria; UCL Institute of Health Informatics,<br>Cassell, Jackie; Brighton and Sussex Medical School, Department of Primary Care and Public Health; UK Health Security Agency<br>Cadar, Dorina; Brighton and Sussex Medical School, Department of Primary Care and Public Health; Brighton and Sussex Medical School, Department of Neuroscience<br>Henderson, Catherine; LSE<br>Knapp, Martin; LSE<br>Goscé, Lara; University College London<br>Leiser, Ruth; University of Strathclyde<br>Regan, Martyn; UK Health Security Agency; University of Manchester<br>Cullen-Stephenson, Iona; UCL, Comprehensive Clinical Trials Unit<br>Fenner, Robert; UCL, Comprehensive Clinical Trials Unit<br>Verma, Arpana; University of Manchester, Division of Population Health, Health Services Research & Primary Care, School of Health Sciences<br>Gordon, Adam; University of Nottingham, Division of Rehabilitation and Ageing; NIHR Applied Research Collaboration-East Midlands (ARC-EM)<br>Hopkins, Susan; UK Health Security Agency<br>Copas, Andrew; UCL, Institute for Global Health<br>Freemantle, Nick; University College London, Comprehensive Clinical Trials Unit<br>Flowers, Paul; University of Strathclyde<br>Shallcross, Laura; UCL
Keywords:	COVID-19, Epidemiology < TROPICAL MEDICINE, Public health < INFECTIOUS DISEASES

For peer review only - http://bmjopen.bmj.com/site/about/guidelines.xhtml

Title: Shaping care home COVID-19 testing policy: A pragmatic cluster randomised controlled trial of asymptomatic testing compared to standard care in care home staff (VIVALDI-CT).

Authors: Natalie Adams[1]; Oliver Stirrup[2]; James Blackstone[3]; Maria Krutikov[1]; Jackie Cassell[4,5]; Dorina Cadar[4,6]; Catherine Henderson[7]; Martin Knapp[7]; Lara Goscé[2,8]; Ruth Leiser[9]; Martyn Regan[5,10,11]; Iona Cullen-Stephenson[3]; Robert Fenner[3]; Arparna Verma[10]; Adam L Gordon[12,13]; Susan Hopkins[5]; Andrew Copas[2]; Nick Freemantle[3]; Paul Flowers[9] & Laura Shallcross[1].

1. Institute of Health informatics, UCL, London, UK
2. Institute for Global Health, UCL, London, UK
3. Comprehensive Clinical Trials Unit, Institute of Clinical Trials and Methodology, UCL, London, UK
4. Department of Primary Care and Public Health, Brighton and Sussex Medical School, Brighton, UK
5. UK Health Security Agency, London, UK
6. Centre for Dementia Studies, Department of Neuroscience, Brighton and Sussex Medical School, Brighton, UK
7. Care Policy and Evaluation Centre, LSE, London, UK
8. Department of Infectious Disease Epidemiology, London School of Hygiene and Tropical Medicine, London, UK
9. School of Psychological Sciences and Health, University of Strathclyde, Glasgow, UK
10. Division of Population Health, Health Services Research & Primary Care, School of Health Sciences, University of Manchester, Manchester, United Kingdom
11. Manchester Academic Health Science Centre, University of Manchester, Manchester, United Kingdom.
12. Academic Unit of Injury, Recovery and Inflammation Sciences (IRIS), School of Medicine, University of Nottingham, Nottingham, UK
13. NIHR Applied Research Collaboration-East Midlands (ARC-EM), Nottingham, UK.

For peer review only - http://bmjopen.bmj.com/site/about/guidelines.xhtml

Corresponding author: Professor Laura Shallcross: l.shallcross@ucl.ac.uk

Word count: 3834/4000

Abstract

Introduction

Care home residents have experienced significant morbidity, mortality and disruption following outbreaks of SARS-CoV-2. Regular SARS-CoV-2 testing of care home staff was introduced to reduce transmission of infection, but it is unclear whether this remains beneficial. The aim of this trial is to investigate whether use of regular asymptomatic staff testing, coupled with funding to reimburse sick pay for those who test positive and meet costs of employing agency staff, is a feasible and effective strategy to reduce the impact of COVID-19 in care homes.

Methods and analysis

The VIVALDI-Clinical Trial (VIVALDI-CT) is a multi-centre, open label, cluster randomised controlled, phase III/IV superiority trial in up to 280 residential and/or nursing homes in England providing care to adults aged >65 years. All regular and agency staff will be enrolled, excepting those who opt out. Homes will be randomised to the intervention arm (twice weekly asymptomatic staff testing for SARS-CoV-2) or the control arm (current national testing guidance). Staff who test positive for SARS-CoV-2 will self-isolate and receive sick pay. Care providers will be reimbursed for costs associated with employing temporary staff to backfill for absence arising directly from the trial.

The trial will be delivered by a multi-disciplinary research team through a series of five work packages.

The primary outcome is the incidence of COVID-19 related hospital admissions in residents. Health economic and modelling analyses will investigate the cost-effectiveness and cost-consequences of the testing intervention. A process evaluation using qualitative interviews will be conducted to understand intervention roll out and identify areas for optimisation to

For peer review only - http://bmjopen.bmj.com/site/about/guidelines.xhtml

inform future intervention scale-up, should the testing approach prove effective and cost effective. Stakeholder engagement will be undertaken to enable the sector to plan for results and their implications and to coproduce recommendations on the use of testing for policymakers.

Strengths and limitations of this study

- VIVALDI-CT will provide the first evidence from a clinical trial to assess the impact of regular asymptomatic SARS-CoV-2 testing of staff to prevent infection and related healthcare outcomes in residents.
- The process evaluation and economic analyses will provide valuable insights into the wider impacts of testing and outbreaks on care home residents and staff, informing policy on the future use of testing for SARS-CoV2 and other pathogens.
- The researchers have previously demonstrated that it is feasible to set-up and deliver large scale studies in care homes on short timescales by working in partnership with care providers. Replicating this approach in a trial represents a new, agile model for the rapid delivery of policy-relevant research, and appeal to care home stakeholders who are frustrated by traditional research timelines.
- Inclusive participation will be a focus by ensuring larger and smaller care groups are included in trial, as well as focusing on diverse settings.

Keywords

COVID-19; SARS-CoV-2; care homes; long-term care facility; epidemiology; asymptomatic testing; randomised controlled trial; public health; policy

For peer review only

For peer review only - http://bmjopen.bmj.com/site/about/guidelines.xhtml

Introduction

Context

In England, approximately 380,000 people (4% of > 65-year-olds) live in 11,000 care homes for older adults. Most care home residents ('residents') are older than 85 years, at least two-thirds live with dementia, and over half die within 12 months of admission to a care home.[1,2] Residents worldwide have experienced among the highest rates of COVID-19 mortality and morbidity,[3] and in England, they have also been subject to particularly strict and lengthy lock-down measures. Prolonged use of COVID-19 restrictions (e.g. social isolation, visitor restrictions) has had a devastating impact on residents' well-being, and their physical and mental health, for example depriving them of contact with family members in their final weeks of life.[4]

Current knowledge

Public health disease control measures were deployed rapidly and simultaneously in care homes early in the pandemic to reduce infection spread, limiting any assessment of the impact of individual measures. There have been no interventional studies of non-pharmaceutical control measures to reduce COVID-19 infection in care homes, but a Cochrane rapid review (published in September 2021) identified 11 observational and 11 modelling studies, all from high-income countries.[5] The review grouped interventions into entry regulations (e.g. reducing visitors), contact regulating and transmission-reducing measures (e.g. Personal Protective Equipment, PPE), surveillance (symptomatic and asymptomatic testing), and outbreak control measures. Across these domains the quality of evidence was poor. In addition, there was widespread recognition that some of these measures, such as preventing visitors from entering the care home, were associated with significant harm.

Throughout the pandemic, testing has been used in three ways to reduce transmission of infection: 1) symptomatic testing, 2) testing during outbreaks to reduce their duration and severity and 3) regular, asymptomatic testing.

For peer review only - http://bmjopen.bmj.com/site/about/guidelines.xhtml

In the UK, compliance with regular testing may have been driven by national policies incentivising testing, including with financial support (e.g., Adult Social Care Rapid Testing Fund introduced in January 2021, Infection Control Fund introduced May 2020).[6,7] However, relatively few published studies have examined how these influence compliance with asymptomatic testing in care homes. We conducted a rapid systematic review, spanning January 2020 to July 2022. It highlighted 14 international papers,[8–21] published in English. No studies used an experimental design, and none reported, or evaluated, interventions designed to improve compliance with SARS-CoV-2 testing. The papers used a range of designs (e.g., qualitative, cross-sectional quantitative, consensus building). Together these studies highlight the *multi-levelled* factors that have shaped adherence with SARS-CoV-2 testing in care homes. We then used the behaviour change wheel[22] as an approach to develop systematically potentially useful intervention content from the factors influencing testing identified within the literature. Subsequently, through a series of stakeholder engagement events with diverse care home staff and representatives from the care home sector, we agreed the content of a multi-level intervention designed to maintain compliance with twice weekly LFD testing for COVID-19 within intervention care homes ('Test to Care').

There remains a lack of evidence on whether the benefits of regular testing for COVID-19 outweigh its harms, and if so, under which scenarios. There have been no attempts in the literature to consolidate the considerable expertise and learning on how to ensure compliance with testing in this setting. From a policy perspective, the key question remains over appropriate thresholds for turning testing 'on' and 'off' in response to varying levels of 'COVID-19 threat' (e.g., high/low levels of infection in the community; the emergence of novel COVID-19 variants).

We posit that the best approach to address these questions is through a randomised clinical trial. Randomisation overcomes the problem of substantial heterogeneity among care homes as they vary in resident population, care provision, and uptake of control measures such as vaccination in staff, or use of facemasks, which limit the conclusions that can be drawn from observational studies. Although there are significant challenges associated with undertaking a trial in care homes in a changing policy and epidemiological context, there is

For peer review only - http://bmjopen.bmj.com/site/about/guidelines.xhtml

an urgent need for high-quality evidence to inform the future use of testing for SARS-CoV-2 and potentially other infections in this setting.

Study aims

We will investigate whether continued use of regular asymptomatic testing in staff is a feasible, effective, and cost-effective strategy to reduce the impact of COVID-19 in care homes. Findings will inform testing policy across the United Kingdom (UK) for COVID-19 and add to knowledge on the use of testing in care homes to prevent other respiratory viruses, such as influenza. These objectives will be delivered through a series of five interlinked work-packages (WPs) which are described in detail in Table 1.

Methods and analysis

Study Design

VIVALDI-CT is a multi-centre, open label, cluster randomised controlled, phase III/IV superiority trial.

Each eligible care home will be randomised to either standard care (SARS-CoV-2 testing policy for care home staff that is in place nationally at the time of trial operation), or regular asymptomatic testing of care home staff for COVID-19 using Lateral Flow Devices (LFDs) combined with support payments for sickness absence and agency staff backfill.

Study Setting

VIVALDI-CT will take place in up to 280 residential and/or nursing homes in England providing care to adults aged >65 years.

Recruitment

Due to rapid timescales for trial delivery, and the need to streamline and centralise data collection we will primarily partner with providers that manage multiple care homes. We will first contact the senior management teams of providers that we have previously worked with in the Vivaldi study[23] to determine if they are interested in trial participation. Providers

For peer review only - http://bmjopen.bmj.com/site/about/guidelines.xhtml
For peer review only

will be asked to supply a list of eligible care homes and confirm that the care home manager has provided consent for each listed home to participate. If we are unable to recruit sufficient homes from the Vivaldi network, we will work with provider representative organisations (e.g., National Care Forum, Care England, National Care Association) to identify other eligible providers.

Homes will be selected to capture diversity in care home size, population (nursing / residential / dementia care), ethnicity, geographical location, rural/urban and provider type (for-profit / not-for-profit). Inclusive participation will be a focus by ensuring larger and smaller care groups are included in trial, as well as focusing on diverse settings.

Inclusion and Exclusion Criteria

Only care home staff are eligible to participate in the testing intervention. This includes temporary (agency) staff with no restrictions i.e., catering, administrative, and maintenance staff, in addition to those in a resident-facing role. However, all care home staff, as well as residents, visitors, and relatives, are eligible to participate in interviews undertaken as part of the trial's process evaluation. All care home residents at participating homes are eligible for data collection and analysis of the outcomes specified.

Visitors, residents, and relatives are not eligible to take part in the testing intervention. Staff who visit the care home to provide care but are not employed by the care home e.g., GPs, health visitors, are not eligible to take part in either the interviews or the testing intervention.

Primary outcome

The primary outcome is the incidence of COVID-19 related hospital admissions in residents defined as admissions with a relevant ICD-10 code (COVID hospitalisations to be defined as any hospital admission record with a primary or secondary ICD10 code of 'U071') and/or admissions in residents who test positive for COVID-19 within 24 hours following admission or in the 7 days before hospital admission. This is considered the most important outcome for policymakers.

Secondary outcomes

For peer review only - http://bmjopen.bmj.com/site/about/guidelines.xhtml

Although we have adopted a healthcare/NHS perspective for the primary outcome, we recognise the importance of capturing outcomes that are relevant to the social care sector, such as outbreaks and care home closures. This is reflected in our choice of secondary outcomes, which include:

- Incidence rate of hospital admissions (all-cause) in residents for non-elective care, measured as events per 100,000 person-days of follow-up over the duration of the trial.
- Incidence rate of COVID-associated mortality in residents, measured as events per 100,000 person-days of follow-up over the duration of the trial.
- Incidence of all-cause mortality in residents, measured as events per 100,000 person-days of follow-up over the duration of the trial.
- Testing uptake in staff, measured as proportion of staff at each home participating in testing during each week of the trial.
- Prevalence of SARS-CoV-2 among staff who test, measured as proportion of staff with positive test result among those with at least one test recorded during each week of the trial.
- Incidence rate of SARS-CoV-2 infections detected in residents, measured as events per 100,000 person-days of follow-up over the duration of the trial.
- Incidence rate of home-level outbreaks, measured as events per 1000 days of follow-up over the trial duration.
- Duration of outbreaks, measured as days from first to last case within outbreaks occurring within the trial period.
- Incidence rate of care home closures due to outbreaks, measured as events per 1000 days of follow-up over the duration of the trial.
- Proportion of staff per home who are off sick during each week of the trial.
- Proportion of all shifts filled by agency staff at each home each week.
- Costs per test.
- Testing metrics e.g., staff time taken to conduct the test at work.
- The impact of testing on resident, staff and visitors e.g., Social Care related Quality of Life collected via interviews in WP3.

For peer review only - http://bmjopen.bmj.com/site/about/guidelines.xhtml

Sample Size

Based on observational data from the VIVALDI study we found that over the 3-month period of January-March 2022 1.8% of residents had a COVID-19 related hospital admission, and the Intra-Cluster Correlation (ICC) across homes was 0.003 (95% CI 0.000-0.007). We assume that we will observe a cumulative incidence of around 3.0% in the trial, which would require a trial duration of 5-6 months if the incidence rate is similar to that in winter 2021/2022, in combination with a conservative ICC value of up to 0.01 (higher in line with the higher cumulative incidence compared to 3 months), and an average care home size of 35 residents with coefficient of variation in size of 0.5. With a total of 280 homes randomised 1:1 to trial arms and taking the usual two-sided test at 5% significance level, the design provides 84% power to detect a reduction in COVID-19 related admissions due to intervention to 1.9% (relative risk 0.63).

Timeline

The trial programme will run from November 2022 to April 2024. The recruitment of care home providers and operation of the intervention will take place between December 2022 and March 2023, with the possibility that this will be extended if deemed necessary for data collection. Table 2 shows a participant timeline and Figure 1 is a schematic of the trial pathway.

Intervention allocation

Care homes will be randomised on a 1:1 ratio. If all providers are ready for trial participation at the same time, then all participating homes will be randomised at the same time. Otherwise, the homes from different providers will be randomised in a phased approach, as they become ready. Randomisation will be performed by the trial statistician based on pseudo-random number generation after trial enrolment and before intervention implementation. Restricted randomisation (specifically covariate constrained randomisation) will be used to ensure balance on care home provider, size, and region.

For peer review only - http://bmjopen.bmj.com/site/about/guidelines.xhtml

Blinding

Researchers and staff of participating care homes will not be blinded to their intervention allocation, as this would not be feasible.

Data collection

To facilitate trial set-up and minimise the burden on care home staff, much of the data for analysis will be obtained from routinely collected healthcare information held within the UK COVID-19 Datastore.[44] This will include results of LFD and PCR tests for SARS-CoV-2 for staff and residents, information on hospital admissions and deaths for residents, and vaccination history for residents. Data within the COVID-19 Datastore are linked to a pseudonymised ID at the level of each individual, which can be linked to Care Quality Commission-ID (CQC-ID), a unique ID number provided by the CQC to identify each care home, for participating care homes and associated staff or resident status. A new study specific pseudonymised ID will be created for each individual before export of data to UCL, in order to prevent any theoretical possibility of reidentification.

Hospital admissions data are linked to ICD-10 diagnostic codes (including COVID-19 codes) however, there is a lag of several months in the assignment of these codes. To allow timely monitoring of data quality for the primary outcome and limit the risk of omitting or double-counting hospital admissions in residents, during the intervention period providers will be asked to upload weekly lists of COVID-associated hospital admissions in residents from participating care homes to the COVID-19 Datastore. Linkage to individual pseudonymised IDs will allow comparison to the routinely collected hospital admission data once available.

To inform estimates of incidence rates which form the primary and secondary outcomes, we will collect the total number of residents in the home on a weekly basis from providers as this will allow us to estimate the denominator. We will also collect the total number of staff on a weekly basis from each home to inform estimates of testing uptake and explore the feasibility of collecting data on the number of staff who opt-out of asymptomatic testing (in the intervention arm).

Care home level (aggregate) data will be collected from providers on dates of care home closures, use of disease control measures, staff sickness absence and employment of agency

For peer review only - http://bmjopen.bmj.com/site/about/guidelines.xhtml

staff to inform health economic analyses. We will explore the feasibility of collecting care home level data on fees paid by residents who are funded by the local authority, and whether it is feasible to collect more detailed information on healthcare utilisation, such as primary care consultations and use of antivirals in residents.

Data on outbreak events (dates, size) will be obtained from the United Kingdom Health Security Agency (UKHSA) Adult Social Care Team. Data on the local incidence of COVID-19 and co-circulation of other respiratory viruses will be obtained from the UKHSA and/or the Office of National Statistics (ONS) Covid Infection Survey.

Data management

Individual-level trial data will be stored in the UCL Data Safe Haven[24] which is hosted by UCL. All identifiable data will be held only by individual care homes or NHS England (NHSE), who will act as Data Processor on behalf of UCL. These databases are protected by multi-layer firewalls with full data encryption at rest and in transit.

For qualitative interviews, upon completion of transcription, the pseudo-anonymised data will be stored on the University of Strathclyde network in a secure, restricted access folder. Consent forms obtained via interviews and focus groups in the process evaluation will also be stored securely.

Statistical analysis

Analysis of the primary outcome, and secondary outcomes expressed as event incidence, will be based on Poisson or negative binomial regression with cluster-robust standard errors, adjusting for calendar time and key care home characteristics used in the restricted randomisation such as provider, region, and size. We will also explore whether the intervention effect differs according to care home size, and other characteristics such as proportion of temporary staff. Unadjusted effect estimates from these analyses will be reported for completeness. Using interaction terms, we will explore whether the effect of the intervention on the primary outcome differed between time periods defined by the national recommendations for testing in the routine care arm, should these change during data collection.

For peer review only - http://bmjopen.bmj.com/site/about/guidelines.xhtml

Analysis of the primary outcome will include all trial care homes (intention to treat analysis), and so represent a treatment policy estimate. We will also define an implementation score based on the frequency and proportion of staff testing at each home based on data the homes provide, which may vary over time. As an exploratory analysis we will assess whether the primary outcome is associated with this implementation score within the intervention arm and express the effect of the intervention relative to control arm for different levels of implementation. This analysis will be based on the same regression method as used for the primary analysis.

Health Economic Analysis

The health economic analysis will investigate the cost-effectiveness and cost-consequences of the testing intervention taking a NHS, Personal Social Services, and a societal perspective. The within-trial costs and outcomes in intervention and control groups will be examined from each perspective. Cost-effectiveness of the intervention in terms of the primary outcome and in terms of all-cause mortality will also be examined. Costs of admission will be excluded from the total costs under consideration in this case.

We will also examine cost-effectiveness in terms of the secondary outcomes of cases prevented and resident deaths prevented, and outcomes of hospital admission and number of outbreaks alongside costs offset/additional costs incurred in a cost-consequences analysis.

Process Evaluation

There is major diversity across care homes, for example in terms of provision of care, resident population, care home size, and the care home workforce. As a result, it is essential to consider the feasibility and sustainability of the intervention and how contextual factors might impact on the ability to scale it if the trial suggests it is effective and cost-effective. These issues will be addressed in the process evaluation which aims to understand intervention roll out and identify areas for optimisation to inform future intervention scale-up, should the testing approach prove effective and cost effective.

The objectives of the process evaluation are:

For peer review only - http://bmjopen.bmj.com/site/about/guidelines.xhtml

- To determine intervention acceptability.
- To determine the role of context in shaping the way, the intervention operated.
- To determine what can be learned about intervention fidelity and adaptation.
- To determine which intervention components worked as anticipated and which need further modification.
- To investigate unanticipated intervention effects.
- To determine what can be learned from the control group.

The process evaluation will develop implementation guidance and training packages ready for future scale up as well as details of minimal care home requirements and staff competencies necessary for intervention delivery.

Qualitative data will be collected from 28 (10%) care homes evenly distributed across each intervention and control arms and spaced across time.

Ethics and dissemination

Study monitoring

An independent Trial Steering Committee (TSC) and Data Monitoring and Ethics Committee (DMEC) will be formally responsible for programme oversight, ensuring the study is conducted in compliance with regulations. The DMEC will also be responsible for monitoring the accumulating data and making recommendations to the TSC on whether the trial should continue as planned.

A Trial Management Group (TMG) will be responsible for the design, coordination, and strategic management of the trial.

Safety reporting

Staff at the sites randomised to asymptomatic testing will report the occurrence of Serious Adverse Events (SAEs) considered 'related' to the intervention only. In such cases site personnel will complete a Serious Adverse Event (SAE) report within 24 hours of notification

For peer review only - http://bmjopen.bmj.com/site/about/guidelines.xhtml
For peer review only

of the event. Clinical review of any SAEs will take place and be reported to the Research Ethics Committee if deemed both ‘related’ to the trial intervention and ‘unexpected.’

Patient and Public Involvement

Patient public involvement (PPI) has already informed the development of this programme, by highlighting the barriers to testing and the need to capture its adverse impacts on staff, residents, and providers. Public advisors have also emphasised the importance of developing a strong plan for implementation, recognising the financial implications of long-term use of testing and sickness payments, informing our emphasis on implementation in WP5.

The PPI team will deliver the following objectives:

- To ensure that the ‘voice and views’ of the public regarding regular testing for COVID-19 are heard by the research team and the wider stakeholder group.
- To create an open, inclusive culture enabling effective communication between the study team, PPI group and the wider stakeholder and oversight groups.
- To agree an approach to communicate outputs from the trial to different audiences, including care home staff, residents and their families and the public using a variety of media.

Research ethics approval

The study has been approved by the London - Bromley Research Ethics Committee (reference number 22/LO/0846) and the Health Research Authority (HRA) (22/CAG/0165).

Consent and opt out

Care home providers and home managers will be asked if their care home(s) are willing to participate in the trial. Individual staff members will have the option to opt out of testing however, high staff turnover, in conjunction with the large number of care homes participating in the trial, means that it is not feasible to obtain individual consent from staff or regarding the use of testing data. Identifiable data submitted by care homes as part of the study will be pseudonymised by NHS England before it is provided to the research team.

For peer review only - http://bmjopen.bmj.com/site/about/guidelines.xhtml

This study has section 251 support to allow the disclosure of confidential patient information (regarding testing in staff) from care homes to NHS England, for the purposes of monitoring uptake of the testing in the control and intervention arms of the trial.

The study will also collect limited individual-level identifiable data from residents to ensure the primary outcome can be determined accurately. It is not feasible to seek individual-level consent from every resident for the use of these data due to high levels of cognitive impairment in residents and excluding data from a large proportion of residents would compromise the scientific value of the trial and the subsequent generalisability of trial findings. This study has section 251 support to allow the disclosure of confidential patient information (regarding residents admitted to hospital) from care homes to NHS England, for the purposes of linkage to the COVID-19 datastore and to enable NHS England to use confidential patient information from SARS-CoV-2 tests to link to other NHS datasets within the COVID-19 datastore. Staff and residents have the option of opting out from the processing and analysis of their individual-level data within this study at any time during the study.

Care home managers in the subset of homes selected for qualitative data collection (focus groups or one to one interviews) will be asked to disseminate recruitment materials to staff within the home via word of mouth, email, or other routine modes of communication. Upon receipt of staff contact details, interested staff will then be sent participant information sheets about the study, given the option to ask questions about the study, complete on-line consent forms and provide brief sociodemographic details to enable the study team to monitor total sample composition. Having checked on-line consent has already been given and after exploring any remaining unanswered questions raised by the PIS, the participants will be asked to also give recorded oral consent to participate.

Confidentiality

All data will be handled in accordance with the Data Protection Act 2018, the UK General Data Protection Regulation (UK GDPR) and subsequent updates and amendments.

For peer review only - http://bmjopen.bmj.com/site/about/guidelines.xhtml

For peer review only

Dissemination policy

The results of the trial will be disseminated regardless of the direction of effect. The publication of the results will comply with a trial-specific publication policy and will include submission to open access journals.

A lay summary of the results will also be produced to disseminate the results to participants. A summary of results will be included online in the publicly accessible HRA website within 12 months of date of trial closure. A Statistical Analysis Plan will also be published under open access arrangements.

Trial Registration and Reporting Guidelines

The VIVALDI-CT was registered with the International Standard Randomised Controlled Trial Number website (ISRCTN 13296529)[25] on 5 December 2022 and the protocol adheres to the SPIRIT 2013 Statement [26].

Author Statement

The study concept and design were conceived by LS, PF, AC, OS, SH.
ICS, LS and RF will conduct data collection. Analysis will be performed by OS, RL, PF, DC, CH, LG, MK and AC
NA, LS and JB prepared the first draft of the manuscript. All authors provided edits and critiqued the manuscript for intellectual content.

Funding

This work is supported by the NIHR Health and Social Care Delivery Research (HSDR) Programme number [154310]. Costs associated with SARS-CoV-2 testing including support payments for care home staff and for care homes to fund agency staff backfill will be funded by the UK Health Security Agency (UKHSA).

Sponsor

The VIVALDI-CT is sponsored by University College London, represented by the UCL Comprehensive Clinical Trials Unit (UCL CCTU).

For peer review only - http://bmjopen.bmj.com/site/about/guidelines.xhtml

Competing interests

None declared.

Study Status

Current Protocol – Version 3.0 dated 04 Jan 2023. At the time of protocol submission, recruitment of sites had been completed.

Data Statement

No data are associated with this article.

Data from the trial will be available for sharing between the trial researchers as detailed in the Data Sharing Agreement between the institutions hosting the VIVALDI-CT researchers.

Requests for access to trial data will be considered, and approved in writing where appropriate, after formal application to the TSC. Considerations for approving access are documented in the TSC Terms of Reference.

Acknowledgements

We are grateful to the care homes who have already agreed to participate in the VIVALDI-CT. We would also like to acknowledge the role of the care homes participating in the VIVALDI Study for sharing their learning from the pandemic with us.

We also acknowledge the support of the independent members of the Trial Steering Committee (TSC):

- Prof Alistair Hay (University of Bristol)
- Dr Jennifer Thompson (LSHTM)
- Dr Michael Larkin (Aston University)
- Zoe Fry (Outstanding Society)
- Samantha Crawley (Bracebridge)
- Margaret Ogden (independent PPI member)

As well as the Data Monitoring and Ethics Committee (DMEC):

- Prof Karla Hemming (University of Birmingham)

For peer review only - http://bmjopen.bmj.com/site/about/guidelines.xhtml

- Dr Tania Kalsi (Guy's and St Thomas' NHS Foundation Trust)
- Dr Terry Quinn (University of Glasgow)

We would also like to thank members of the UKHSA:

- Dr Tom Fowler (UKHSA)
- Alex Barton (DHSC)
- Prof Jackie Cassell (UKHSA)
- Dr Sarah Tunkel (UKHSA)

For peer review only

For peer review only - http://bmjopen.bmj.com/site/about/guidelines.xhtml

Figures and tables

Table 1: Description of work packages in the VIVALDI-CT

Work Package	Aims	Participants	Methods	Data collection	Data analysis	Outcomes
WP1	To coproduce a sustainable testing intervention for care home staff.	Care home staff, providers and policymakers.	A series of workshops with care home stakeholders (e.g., home managers, staff, providers) and policymakers (including those with knowledge of testing logistics).<br><br>A workshop will also take place with residents with capacity to consent, relatives and visitors.	1) Consolidation of existing insights into routine testing gleaned through past experiences with the COVID-19 pandemic; 2) discussion of the intervention prototype; and 3) Operationalisation of intervention in ways which are likely to be acceptable and appropriate within the sector.	Data (transcript, detailed notes, images) will be analysed thematically to further develop the initial programme theory specifying important elements of the context, key intervention components, their mechanisms, and their relation to a hierarchy of outcome measures (e.g., health, social and financial outcomes).	Development of a testing intervention for use in WP2
WP2	To evaluate the effectiveness of the testing intervention compared to the recommended testing protocol that is in place at the time in a pragmatic cluster randomised trial.	Care home staff.	A cluster randomised controlled trial in which asymptomatic care home staff will be randomised at provider level to either test twice weekly using LFDs or to follow current national testing guidance.	At enrolment, providers will complete a checklist summarising characteristics of each participating care home. Each week during the testing period care home managers will be asked to record: 1) dates of hospital admissions for residents and their corresponding NHS	Analysis of the primary outcome, and secondary outcomes expressed as event incidence, will be based on Poisson or negative binomial regression with cluster-robust standard errors, adjusting for calendar time and key care home characteristics used in the restricted	The primary outcome will be the incidence of COVID-19 related hospital residents.

For peer review only - http://bmjopen.bmj.com/site/about/guidelines.xhtml

				numbers and/or identifiers, 2) number of staff and residents in each care home. The study will collect limited individual-level identifiable data from residents to ensure that the primary outcome of hospital admissions is accurate. Linkage to other routine datasets (hospitalisations, cause of death, COVID-19 test results, vaccination status).	randomisation such as provider, region, and size.	
WP3A	To understand the intervention roll out and identify areas for optimisation to inform future intervention scale-up, should the testing approach prove effective and cost effective	Care home staff	The intervention programme theory and associated logic models from WP1 will be used to undertake a parallel mixed methods process evaluation.<br>Interviews and focus groups will be undertaken with staff.	Data collection will be facilitated though virtual platforms (according to participant norms).<br><br>Quantitative data will include descriptive statistics from across all trial sites complemented by multivariate analyses of data sets where appropriate.<br><br>Qualitative data will be collected from 28 (10%) care homes evenly distributed across each	A combination of deductive and inductive thematic analysis will be used.<br><br>Interview/focus group transcriptions and qualitative survey data will be imported into NVivo10 software to facilitate data handling, organisation and coding. Analysis will firstly be thematic and use a combination of deductive and inductive thematic analysis.	Implementation guidance and training packages ready for future scale up;<br><br>Details of minimal care home requirements and staff competencies necessary for intervention delivery.

For peer review only - http://bmjopen.bmj.com/site/about/guidelines.xhtml

				intervention and control arms and spaced across time. Selection criteria will focus on geography, socio-economic status of area, highest/lowest rates of infection. Heterogeneous samples of staff will be recruited. After consent is attained qualitative data will be collected primarily using focus groups. One to one interviews will also be possible if requested.		
WP3B	To conduct a mixed methods, process evaluation and exploratory analysis to explore the impact of testing and outbreaks on social care related quality of life in home care residents	Care home residents	The Adult Social Care Outcomes Toolkit (ASCOT) will be used to assess the impact of the intervention and outbreaks on residents. ASCOT is a well-established tool for measuring social care related quality of life (SCRQoL). We will establish an ASCOT cohort of six care homes stratified by type and size, distributed equally across control and intervention arms. Homes taking part in the	In person interviews. If in-person resident interviews are not possible, Essential Caregivers (designated visitor allowed even during outbreaks) or members of staff will be interviewed.	The ASCOT tool will be used to assess SCRQoL at baseline, during/immediately after an outbreak or mid study, and at end of the study. ASCOT data will be combined with individual level demographic and health and aggregate home level data to give a comprehensive description of SCRQoL.	Outputs will also be integrated with findings from WP3A to provide a holistic assessment of the acceptability and feasibility of the testing intervention.

For peer review only - http://bmjopen.bmj.com/site/about/guidelines.xhtml

			qualitative interviews in WP3A are excluded.			
WP4A	To evaluate the costs and benefits of the testing intervention.	NHS, providers, residents, families and staff.	We will examine within-trial costs and outcomes in intervention and control groups from each perspective. We will examine the cost-effectiveness of the intervention in terms of the primary outcome and in terms of all-cause mortality. The costs of admission will be excluded from the total costs under consideration in this case. We will also examine cost-effectiveness in terms of the secondary outcomes of cases prevented and resident deaths prevented. We will examine the outcomes of hospital admission and number of outbreaks alongside costs offset/additional	We will examine the cost-effectiveness of the intervention in terms of the primary outcome, excluding cost of admission from total cost. We will also examine cost-effectiveness in terms of secondary outcomes of cases prevented and resident deaths prevented. We will examine outcomes of hospital admission and number of outbreaks alongside costs offset/additional costs incurred in a cost-consequences analysis. investigate the cost-effectiveness and cost-consequences of the testing intervention taking both a NHS, Personal Social Services, and a societal perspective. We will also explore the short and long-term costs of the	Generalised linear models appropriate to counts (hospital admissions, numbers of outbreaks, cases) or binary outcomes (deaths) and costs will be applied. For the purposes of the cost-effectiveness analyses, these will take into account possible correlations between costs and outcomes either by non-parametric bootstrapping of separate regressions or joint modelling approaches such as seemingly unrelated regressions. Where individual level data are available, analyses will take a multilevel approach to adjust for clustering at the care home level either by two-stage bootstrapping	Cost-effectiveness of the intervention in terms of the primary trial outcome (incidence of COVID-19 related hospital admission in residents.)<br><br>Cost-effectiveness in terms of the secondary outcomes of cases prevented and resident deaths prevented.<br><br>Cost-consequences of hospital admission and number of outbreaks alongside costs

For peer review only - http://bmjopen.bmj.com/site/about/guidelines.xhtml

			costs incurred in a cost-consequences analysis.	testing intervention under different epidemiological scenarios (e.g., high/low community incidence of infection).	of separate regressions or simultaneous modelling. Incremental cost-effectiveness ratios will be presented, net benefit calculated over a range of willingness to pay values for gains in outcomes to generate cost-effectiveness acceptability curves.	offset/additional costs incurred.
WP4B	To model costs and benefits of the testing intervention under different scenarios	NHS, providers, residents, families and staff.	A compartmental model will be built to study transmission of infection and infer the proportion of COVID-19 infections and deaths averted by the intervention under different epidemiological scenarios (e.g., high/low community incidence of infection, care home population size etc).<br><br>Two scenarios will be modelled: 1) standard care: residents and staff get tested only if they show symptoms; and 2) intervention: standard	Data from WP4A will be used.	The model will consider two populations i.e., home residents and staff, and take into account both symptomatic and asymptomatic cases, as well as hospital admissions and deaths. The model will be calibrated to trial results and modelling results will be projected in time by extending the time horizon. Unit costs calculated in the first part of the economic analysis will be discounted to future years values and	Estimate of the projected cost-effectiveness of the intervention.

For peer review only - http://bmjopen.bmj.com/site/about/guidelines.xhtml

			care plus regular testing of all staff for COVID-19.		associated to modelling results to estimate the projected cost-effectiveness of the intervention.	
WP5	To coproduce recommendations on the use of regular testing for policymakers	Providers, care home staff, the NHS and primary care, policymakers and public health teams at national and regional / local level, community, residents and families.	Three round-table discussions for a maximum of 20 stakeholders.	Discussions will be based on world café methodological principles. The views of the community, residents and families will be represented through organisations such as Healthwatch, the Residents and Relatives association and the VIVALDI-CT PPI group.	Data (transcript, detailed notes, images) will be analysed thematically.	Formal mechanism to ensure stakeholders are aware and involved in the work as it progresses and to enable the sector to prepare and plan for results and their implications.<br><br>Production of recommendations on the use of regular testing for policymakers.

For peer review only - http://bmjopen.bmj.com/site/about/guidelines.xhtml
For peer review only

Table 2: Participant Timeline

**Trial visit number**	**Baseline**	**Care home data collection period**							
**Month**	**Month 0**	**Months 1-4**		**Survey**	**Routine COVID-19 datastore**	**Uploaded to Foundry by provider data manager**	**To be reported as and when occurs**	**Weekly collection from providers**	**Participants**
		**Sites on Intervention arm**	**Sites on Standard Care arm**						
Demography	x			x	x				
Resident registry (patient type)	x			x					
Vaccination status	x				x				
Care home characteristics	x			x	x				
Number of residents per home (weekly)		x	x					x	
COVID-associated hospital admission events in residents		x	x		x	x			
Number of staff absent from work		x	x					x	
Total shifts at home this week (sum for all staff)		x	x					x	
Number of agency shifts		X	x					x	

For peer review only - http://bmjopen.bmj.com/site/about/guidelines.xhtml

Number of staff per home (weekly)		x	x					x	
Number of staff opting out of asymptomatic testing (weekly)*		x						x	
Routine data on LFD and PCR tests (staff+residents)		x	x		x				
Routine data on hospital admissions (residents)		x	x		x				
Routine data on mortality (residents)		x	x		x				
Outbreak event data		x	x					x	
Safety reporting		x	x				x	x	
Process Evaluation (3a)									
Focus groups		x	x						x
Interviews		x	x						x

* If the control arm starts routine testing, then collected from both arms

For peer review only

For peer review only - http://bmjopen.bmj.com/site/about/guidelines.xhtml

Figure 1: Flow diagram of trial pathway

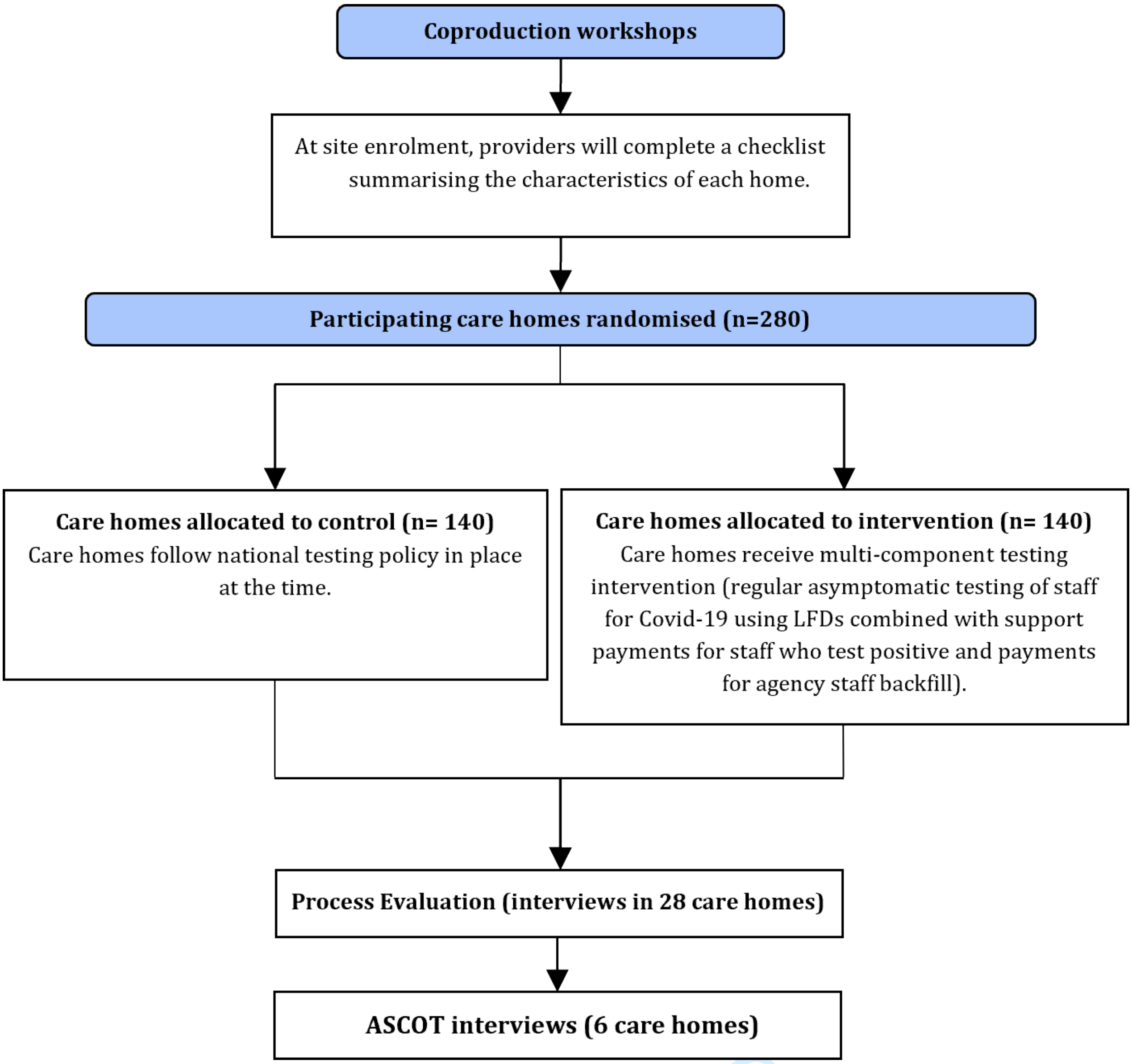

For peer review only - http://bmjopen.bmj.com/site/about/guidelines.xhtml

References

1. Kinley J, Hockley J, Stone L, et al. The provision of care for residents dying in UK nursing care homes. *Age Ageing*. 2014;43(3):375-379. doi:10.1093/ageing/aft158

2. Alzheimer's Society. *Dementia UK Update*.; 2014. Accessed November 27, 2021. https://www.alzheimers.org.uk/sites/default/files/migrate/downloads/dementia_uk_update.pdf

3. Levin AT, Jylhävä J, Religa D, Shallcross L. COVID-19 prevalence and mortality in longer-term care facilities. *Eur J Epidemiol*. 2022;37(3):227-234. doi:10.1007/s10654-022-00861-w

4. Kaelen S, van den Boogaard W, Pellecchia U, et al. How to bring residents' psychosocial well-being to the heart of the fight against Covid-19 in Belgian nursing homes—A qualitative study. Kotozaki Y, ed. *PLOS ONE*. 2021;16(3):e0249098. doi:10.1371/journal.pone.0249098

5. Stratil JM, Biallas RL, Burns J, et al. Non-pharmacological measures implemented in the setting of long-term care facilities to prevent SARS-CoV-2 infections and their consequences: a rapid review. Cochrane Public Health Group, ed. *Cochrane Database Syst Rev*. 2021;2021(9). doi:10.1002/14651858.CD015085.pub2

6. Adult Social Care Rapid Testing Fund. GOV.UK. Accessed October 7, 2022. https://www.gov.uk/government/publications/adult-social-care-rapid-testing-fund

7. Adult Social Care Infection Control Fund. GOV.UK. Accessed July 26, 2022. https://www.gov.uk/government/publications/adult-social-care-infection-control-fund

8. Gray KL, Birtles H, Reichelt K, James IA. The experiences of care home staff during the COVID-19 pandemic: A systematic review. *Aging Ment Health*. 2022;26(10):2080-2089. doi:10.1080/13607863.2021.2013433

9. Pétré B, Paridans M, Gillain N, et al. Factors influencing the adoption and participation rate of nursing homes staff in a saliva testing screening programme for COVID-19. *PloS One*. Published online June 30, 2022. doi:10.1371/journal.pone.0270551

10. Pétré B, Paridans M, Gillain N, et al. Acceptability of Community Saliva Testing in Controlling the COVID-19 Pandemic: Lessons Learned from Two Case Studies in Nursing Homes and Schools. *Patient Prefer Adherence*. 2022;16:625-631. doi:10.2147/PPA.S349742

11. Walker, J. Implementing COVID-19 infection prevention and control measures in long-term care settings. *Nurs Older People*. Published online April 5, 2022. doi:10.7748/nop.2022.e1384

12. Nyashanu M, Pfende F, Ms E. Triggers of mental health problems among frontline healthcare workers during the COVID-19 pandemic in private care homes and

For peer review only - http://bmjopen.bmj.com/site/about/guidelines.xhtml

domiciliary care agencies: Lived experiences of care workers in the Midlands region, UK. *Health Soc Care Community*. Published online February 2022. doi:10.1111/hsc.13204

13. Tulloch J, Micocci M, Buckle P, et al. Enhanced lateral flow testing strategies in care homes are associated with poor adherence and were insufficient to prevent COVID-19 outbreaks: results from a mixed methods implementation study. *Age Ageing*. Published online November 10, 2021. doi:10.1093/ageing/afab162

14. Kierkegaard P, Micocci M, McLister A, et al. Implementing lateral flow devices in long-term care facilities: experiences from the Liverpool COVID-19 community testing pilot in care homes- a qualitative study. *BMC Health Serv Res*. Published online October 25, 2021. doi:10.1186/s12913-021-07191-9

15. Oldfield B, DeCosta S, Petterson L, Lagarde S, Olson D. A Blueprint for Community Health Center and Nursing Home Partnership: Testing for COVID-19 among Residents and Staff at Long-term Care Facilities. *J Health Care Poor Underserved*. Published online 2021. doi:10.1353/hpu.2021.0001

16. Micocci M, Gordon A, Allen A, et al. COVID-19 testing in English care homes and implications for staff and residents. *Age Ageing*. Published online May 5, 2021. doi:10.1093/ageing/afab015

17. White E, Wetle T, Reddy A, Baier R. Front-line Nursing Home Staff Experiences During the COVID-19 Pandemic. *J Am Med Dir Assoc*. Published online January 2021. doi:10.1016/j.jamda.2020.11.022

18. Ouslander J, Grabowski D. COVID-19 in Nursing Homes: Calming the Perfect Storm. *J Am Geriatr Soc*. Published online October 2020. doi:10.1111/jgs.16784

19. Wasserman M, Ouslander J, Lam A, et al. Editorial: Diagnostic Testing for SARS-Coronavirus-2 in the Nursing Facility: Recommendations of a Delphi Panel of Long-Term Care Clinicians. *J Nutr Health Aging*. Published online 2020. doi:10.1007/s12603-020-1401-9

20. Gordon A, Goodman C, Achterberg W, et al. Commentary: COVID in care homes-challenges and dilemmas in healthcare delivery. *Age Ageing*. Published online August 24, 2020. doi:10.1093/ageing/afaa113

21. COVID-19 and policies for care homes in the first wave of the pandemic in European welfare states: Too little, too late? - Mary Daly, Margarita León, Birgit Pfau-Effinger, Costanzo Ranci, Tine Rostgaard, 2022. Accessed July 26, 2022. https://journals.sagepub.com/doi/full/10.1177/09589287211055672

22. Michie S, van Stralen MM, West R. The behaviour change wheel: A new method for characterising and designing behaviour change interventions. *Implement Sci*. 2011;6(1):42. doi:10.1186/1748-5908-6-42

For peer review only

For peer review only - http://bmjopen.bmj.com/site/about/guidelines.xhtml

23. Krutikov M, Palmer T, Donaldson A, et al. Study Protocol: Understanding SARS-Cov-2 infection, immunity and its duration in care home residents and staff in England (VIVALDI). *Wellcome Open Res*. 2021;5:232. doi:10.12688/wellcomeopenres.16193.2

24. UCL. Data Safe Haven (DSH). Information Services Division. Published February 14, 2018. Accessed October 7, 2022. https://www.ucl.ac.uk/isd/services/file-storage-sharing/data-safe-haven-dsh

25. Shallcross L, Shallcross L, Enquiries C. Regular asymptomatic COVID-19 testing in care home staff. doi:10.1186/ISRCTN13296529

26. Chan AW, Tetzlaff JM, Altman DG, et al. SPIRIT 2013 statement: defining standard protocol items for clinical trials. *Ann Intern Med*. 2013;158(3):200-207. doi:10.7326/0003-4819-158-3-201302050-00583

For peer review only

For peer review only - http://bmjopen.bmj.com/site/about/guidelines.xhtml

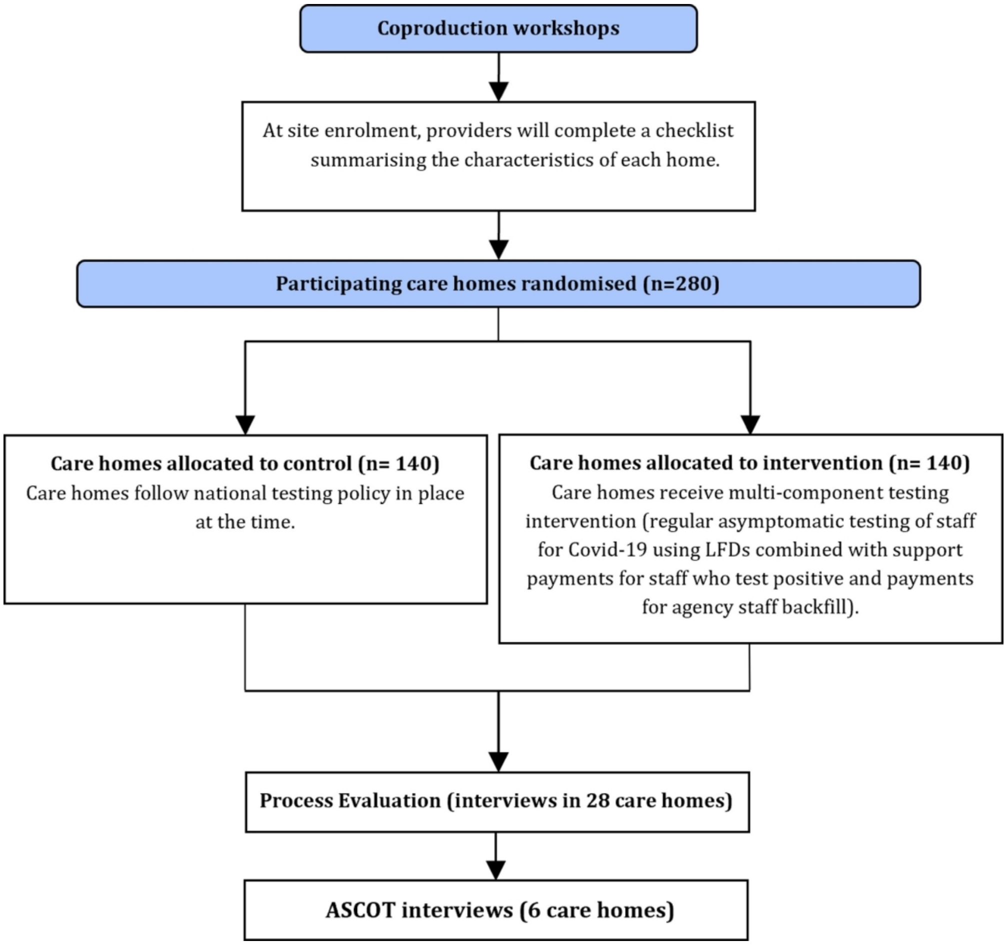

Figure 1: Flow diagram of trial pathway

166x156mm (300 x 300 DPI)

STANDARD PROTOCOL ITEMS: RECOMMENDATIONS FOR INTERVENTIONAL TRIALS

SPIRIT 2013 Checklist: Recommended items to address in a clinical trial protocol and related documents*

Section/item	Item No	Description	Page No
**Administrative information**			
Title	1	Descriptive title identifying the study design, population, interventions, and, if applicable, trial acronym	1
Trial registration	2a	Trial identifier and registry name. If not yet registered, name of intended registry	16
	2b	All items from the World Health Organization Trial Registration Data Set	1-17
Protocol version	3	Date and version identifier	17
Funding	4	Sources and types of financial, material, and other support	16
Roles and responsibilitie s	5a	Names, affiliations, and roles of protocol contributors	16-18
	5b	Name and contact information for the trial sponsor	16
	5c	Role of study sponsor and funders, if any, in study design; collection, management, analysis, and interpretation of data; writing of the report; and the decision to submit the report for publication, including whether they will have ultimate authority over any of these activities	16,17
	5d	Composition, roles, and responsibilities of the coordinating centre, steering committee, endpoint adjudication committee, data management team, and other individuals or groups overseeing the trial, if applicable (see Item 21a for data monitoring committee)	16-18
**Introduction**			
Background and rationale	6a	Description of research question and justification for undertaking the trial, including summary of relevant studies (published and unpublished) examining benefits and harms for each intervention	4-5
	6b	Explanation for choice of comparators	4-5
Objectives	7	Specific objectives or hypotheses	5-6

For peer review only - http://bmjopen.bmj.com/site/about/guidelines.xhtml

Trial design	8	Description of trial design including type of trial (eg, parallel group, crossover, factorial, single group), allocation ratio, and framework (eg, superiority, equivalence, noninferiority, exploratory)	6

Methods: Participants, interventions, and outcomes

Study setting	9	Description of study settings (eg, community clinic, academic hospital) and list of countries where data will be collected. Reference to where list of study sites can be obtained	6
Eligibility criteria	10	Inclusion and exclusion criteria for participants. If applicable, eligibility criteria for study centres and individuals who will perform the interventions (eg, surgeons, psychotherapists)	7
Interventions	11a	Interventions for each group with sufficient detail to allow replication, including how and when they will be administered	9
	11b	Criteria for discontinuing or modifying allocated interventions for a given trial participant (eg, drug dose change in response to harms, participant request, or improving/worsening disease)	13
	11c	Strategies to improve adherence to intervention protocols, and any procedures for monitoring adherence (eg, drug tablet return, laboratory tests)	6
	11d	Relevant concomitant care and interventions that are permitted or prohibited during the trial	6
Outcomes	12	Primary, secondary, and other outcomes, including the specific measurement variable (eg, systolic blood pressure), analysis metric (eg, change from baseline, final value, time to event), method of aggregation (eg, median, proportion), and time point for each outcome. Explanation of the clinical relevance of chosen efficacy and harm outcomes is strongly recommended	7-8
Participant timeline	13	Time schedule of enrolment, interventions (including any run-ins and washouts), assessments, and visits for participants. A schematic diagram is highly recommended (see Figure)	9, Table 2
Sample size	14	Estimated number of participants needed to achieve study objectives and how it was determined, including clinical and statistical assumptions supporting any sample size calculations	9
Recruitment	15	Strategies for achieving adequate participant enrolment to reach target sample size	6

Methods: Assignment of interventions (for controlled trials)

Allocation:

Sequence generation	16a	Method of generating the allocation sequence (eg, computer-generated random numbers), and list of any factors for stratification. To reduce predictability of a random sequence, details of any planned restriction (eg, blocking) should be provided in a separate document that is unavailable to those who enrol participants or assign interventions	9
Allocation concealment mechanism	16b	Mechanism of implementing the allocation sequence (eg, central telephone; sequentially numbered, opaque, sealed envelopes), describing any steps to conceal the sequence until interventions are assigned	9
Implementation	16c	Who will generate the allocation sequence, who will enrol participants, and who will assign participants to interventions	9
Blinding (masking)	17a	Who will be blinded after assignment to interventions (eg, trial participants, care providers, outcome assessors, data analysts), and how	10
	17b	If blinded, circumstances under which unblinding is permissible, and procedure for revealing a participant's allocated intervention during the trial	N/A
**Methods: Data collection, management, and analysis**			
Data collection methods	18a	Plans for assessment and collection of outcome, baseline, and other trial data, including any related processes to promote data quality (eg, duplicate measurements, training of assessors) and a description of study instruments (eg, questionnaires, laboratory tests) along with their reliability and validity, if known. Reference to where data collection forms can be found, if not in the protocol	10-11
	18b	Plans to promote participant retention and complete follow-up, including list of any outcome data to be collected for participants who discontinue or deviate from intervention protocols	10-11
Data management	19	Plans for data entry, coding, security, and storage, including any related processes to promote data quality (eg, double data entry; range checks for data values). Reference to where details of data management procedures can be found, if not in the protocol	11
Statistical methods	20a	Statistical methods for analysing primary and secondary outcomes. Reference to where other details of the statistical analysis plan can be found, if not in the protocol	11-12
	20b	Methods for any additional analyses (eg, subgroup and adjusted analyses)	11-12
	20c	Definition of analysis population relating to protocol non-adherence (eg, as randomised analysis), and any statistical methods to handle missing data (eg, multiple imputation)	10-11

For peer review only - http://bmjopen.bmj.com/site/about/guidelines.xhtml

Methods: Monitoring

Data monitoring	21a	Composition of data monitoring committee (DMC); summary of its role and reporting structure; statement of whether it is independent from the sponsor and competing interests; and reference to where further details about its charter can be found, if not in the protocol. Alternatively, an explanation of why a DMC is not needed	13
	21b	Description of any interim analyses and stopping guidelines, including who will have access to these interim results and make the final decision to terminate the trial	13
Harms	22	Plans for collecting, assessing, reporting, and managing solicited and spontaneously reported adverse events and other unintended effects of trial interventions or trial conduct	13
Auditing	23	Frequency and procedures for auditing trial conduct, if any, and whether the process will be independent from investigators and the sponsor	13

Ethics and dissemination

Research ethics approval	24	Plans for seeking research ethics committee/institutional review board (REC/IRB) approval	14
Protocol amendments	25	Plans for communicating important protocol modifications (eg, changes to eligibility criteria, outcomes, analyses) to relevant parties (eg, investigators, REC/IRBs, trial participants, trial registries, journals, regulators)	16
Consent or assent	26a	Who will obtain informed consent or assent from potential trial participants or authorised surrogates, and how (see Item 32)	14-15
	26b	Additional consent provisions for collection and use of participant data and biological specimens in ancillary studies, if applicable	14-15
Confidentialit y	27	How personal information about potential and enrolled participants will be collected, shared, and maintained in order to protect confidentiality before, during, and after the trial	15
Declaration of interests	28	Financial and other competing interests for principal investigators for the overall trial and each study site	17
Access to data	29	Statement of who will have access to the final trial dataset, and disclosure of contractual agreements that limit such access for investigators	17
Ancillary and post-trial care	30	Provisions, if any, for ancillary and post-trial care, and for compensation to those who suffer harm from trial participation	N/A

For peer review only - http://bmjopen.bmj.com/site/about/guidelines.xhtml

Dissemination policy	31a	Plans for investigators and sponsor to communicate trial results to participants, healthcare professionals, the public, and other relevant groups (eg, via publication, reporting in results databases, or other data sharing arrangements), including any publication restrictions	16
	31b	Authorship eligibility guidelines and any intended use of professional writers	16
	31c	Plans, if any, for granting public access to the full protocol, participant-level dataset, and statistical code	17
**Appendices**			
Informed consent materials	32	Model consent form and other related documentation given to participants and authorised surrogates	N/A
Biological specimens	33	Plans for collection, laboratory evaluation, and storage of biological specimens for genetic or molecular analysis in the current trial and for future use in ancillary studies, if applicable	N/A

*It is strongly recommended that this checklist be read in conjunction with the SPIRIT 2013 Explanation & Elaboration for important clarification on the items. Amendments to the protocol should be tracked and dated. The SPIRIT checklist is copyrighted by the SPIRIT Group under the Creative Commons "Attribution-NonCommercial-NoDerivs 3.0 Unported" license.

For peer review only

BMJ Open

Shaping care home COVID-19 testing policy: A Protocol for a pragmatic cluster randomised controlled trial of asymptomatic testing compared to standard care in care home staff (VIVALDI-CT).

Journal:	*BMJ Open*
Manuscript ID	bmjopen-2023-076210.R1
Article Type:	Protocol
Date Submitted by the Author:	04-Oct-2023
Complete List of Authors:	Adams, Natalie; Royal Free London NHS Foundation Trust; University College London, Institute of Health informatics<br>Stirrup , Oliver ; University College London, Institute for Global Health<br>Blackstone, James; University College London, Comprehensive Clinical Trials Unit<br>Krutikov, Maria; University College London, Institute of Health informatics<br>Cassell, Jackie; Brighton and Sussex Medical School, Department of Primary Care and Public Health; UK Health Security Agency<br>Cadar, Dorina; Brighton and Sussex Medical School, Department of Primary Care and Public Health; Brighton and Sussex Medical School, Centre for Dementia Studies, Department of Neuroscience<br>Henderson, Catherine; The London School of Economics and Political Science, Care Policy and Evaluation Centre<br>Knapp, Martin; The London School of Economics and Political Science, Care Policy and Evaluation Centre<br>Goscé, Lara; University College London, Institute for Global Health; London School of Hygiene & Tropical Medicine, Department of Infectious Disease Epidemiology<br>Leiser, Ruth; University of Strathclyde, Department of Psychological Sciences and Health<br>Regan, Martyn; UK Health Security Agency; The University of Manchester, Division of Population Health, Health Services Research & Primary Care, School of Health Sciences & Manchester Academic Health Science Centre<br>Cullen-Stephenson, Iona; University College London, Comprehensive Clinical Trials Unit<br>Fenner, Robert; University College London, Comprehensive Clinical Trials Unit<br>Verma, Arpana; The University of Manchester, Division of Population Health, Health Services Research & Primary Care, School of Health Sciences<br>Gordon, Adam; University of Nottingham, 12. Academic Unit of Injury, Recovery and Inflammation Sciences (IRIS), School of Medicine; NIHR, Applied Research Collaboration-East Midlands (ARC-EM)<br>Hopkins, Susan; UK Health Security Agency<br>Copas, Andrew; University College London, Institute for Global Health<br>Freemantle, Nick; University College London, Comprehensive Clinical

	Trials Unit<br>Flowers, Paul; University of Strathclyde, Department of Psychological Sciences and Health<br>Shallcross, Laura; University College London, Institute of Health informatics
**Primary Subject Heading**:	Infectious diseases
Secondary Subject Heading:	Epidemiology, Public health
Keywords:	COVID-19, Epidemiology < TROPICAL MEDICINE, Public health < INFECTIOUS DISEASES

SCHOLARONE™
Manuscripts

Title: Shaping care home COVID-19 testing policy: A Protocol for a pragmatic cluster randomised controlled trial of asymptomatic testing compared to standard care in care home staff (VIVALDI-CT).

Authors: Natalie Adams[1]; Oliver Stirrup[2]; James Blackstone[3]; Maria Krutikov[1]; Jackie Cassell[4,5]; Dorina Cadar[4,6]; Catherine Henderson[7]; Martin Knapp[7]; Lara Goscé[2,8]; Ruth Leiser[9]; Martyn Regan[5,10,11]; Iona Cullen-Stephenson[3]; Robert Fenner[3]; Aparna Verma[10]; Adam L Gordon[12,13]; Susan Hopkins[5]; Andrew Copas[2]; Nick Freemantle[3]; Paul Flowers[9] & Laura Shallcross[1].

1. Institute of Health informatics, UCL, London, UK
2. Institute for Global Health, UCL, London, UK
3. Comprehensive Clinical Trials Unit, Institute of Clinical Trials and Methodology, UCL, London, UK
4. Department of Primary Care and Public Health, Brighton and Sussex Medical School, Brighton, UK
5. UK Health Security Agency, London, UK
6. Centre for Dementia Studies, Department of Neuroscience, Brighton and Sussex Medical School, Brighton, UK
7. Care Policy and Evaluation Centre, London School of Economics and Political Science, London, UK
8. Department of Infectious Disease Epidemiology, London School of Hygiene and Tropical Medicine, London, UK
9. Department of Psychological Sciences and Health, University of Strathclyde, Glasgow, UK
10. Division of Population Health, Health Services Research & Primary Care, School of Health Sciences, The University of Manchester, Manchester, United Kingdom
11. Manchester Academic Health Science Centre, The University of Manchester, Manchester, United Kingdom.
12. Academic Unit of Injury, Recovery and Inflammation Sciences (IRIS), School of Medicine, University of Nottingham, Nottingham, UK
13. NIHR Applied Research Collaboration-East Midlands (ARC-EM), Nottingham, UK.

For peer review only - http://bmjopen.bmj.com/site/about/guidelines.xhtml

Corresponding author: Professor Laura Shallcross: l.shallcross@ucl.ac.uk

Word count: 3938/4000

Abstract

Introduction

Care home residents have experienced significant morbidity, mortality and disruption following outbreaks of SARS-CoV-2. Regular SARS-CoV-2 testing of care home staff was introduced to reduce transmission of infection, but it is unclear whether this remains beneficial. This trial aims is to investigate whether use of regular asymptomatic staff testing, alongside funding to reimburse sick pay for those who test positive and meet costs of employing agency staff, is a feasible and effective strategy to reduce COVID-19 impact in care homes.

Methods and analysis

The VIVALDI-Clinical Trial (VIVALDI-CT) is a multi-centre, open label, cluster randomised controlled, phase III/IV superiority trial in up to 280 residential and/or nursing homes in England providing care to adults aged >65 years. All regular and agency staff will be enrolled, excepting those who opt out. Homes will be randomised to the intervention arm (twice weekly asymptomatic staff testing for SARS-CoV-2) or the control arm (current national testing guidance). Staff who test positive for SARS-CoV-2 will self-isolate and receive sick pay. Care providers will be reimbursed for costs associated with employing temporary staff to backfill for absence arising directly from the trial.

The trial will be delivered by a multi-disciplinary research team through a series of five work packages.

The primary outcome is incidence of COVID-19-related hospital admissions in residents. Secondary outcomes include the number and duration of outbreaks and home closures. Health economic and modelling analyses will investigate the cost-effectiveness and cost-

For peer review only - http://bmjopen.bmj.com/site/about/guidelines.xhtml

consequences of the testing intervention. A process evaluation using qualitative interviews will be conducted to understand intervention roll out and identify areas for optimisation to inform future intervention scale-up, should the testing approach prove effective and cost-effective. Stakeholder engagement will be undertaken to enable the sector to plan for results and their implications and to coproduce recommendations on the use of testing for policymakers.

Ethics and dissemination

The study has been approved by the London - Bromley Research Ethics Committee (reference number 22/LO/0846) and the Health Research Authority (HRA) (22/CAG/0165). The results of the trial will be disseminated regardless of the direction of effect. The publication of the results will comply with a trial-specific publication policy and will include submission to open access journals. A lay summary of the results will also be produced to disseminate the results to participants.

Trial registration details

The VIVALDI-CT was registered with the International Standard Randomised Controlled Trial Number website (ISRCTN 13296529) on 5 December 2022.

Strengths and limitations

- First trial to evaluate the benefits and harms of regularly testing care home staff for COVID-19 to protect residents from severe outcomes following infection
- Process evaluation, economic and modelling analyses will provide insights into intervention feasibility and costs/cost-effectiveness, informing future public health policy.
- The study demonstrates the potential for large scale trials in care homes that are delivered in partnership with care providers and capitalise on routinely collected data.
-
- The trial is being delivered in a rapidly changing policy and epidemiological context, which could undermine effective trial delivery.

For peer review only - http://bmjopen.bmj.com/site/about/guidelines.xhtml

Keywords

COVID-19; SARS-CoV-2; care homes; long-term care facility; epidemiology; asymptomatic testing; randomised controlled trial; public health; policy

For peer review only

For peer review only - http://bmjopen.bmj.com/site/about/guidelines.xhtml

Introduction

Context

In England, approximately 380,000 people (4% of > 65-year-olds) live in 11,000 care homes for older adults. Most care home residents (‘residents’) are older than 85 years, at least two-thirds live with dementia, and over half die within 12 months of admission to a care home.(1,2) Care home residents worldwide have experienced among the highest rates of COVID-19 mortality and morbidity,(3) and in England, they have also been subject to particularly strict and lengthy lock-down measures. Prolonged use of COVID-19 restrictions (e.g. social isolation, visitor restrictions) has had a devastating impact on residents’ well-being, and their physical and mental health, for example depriving them of contact with family members in their final weeks of life.(4)

Current knowledge

Public health disease control measures were deployed rapidly and simultaneously in care homes early in the pandemic to reduce infection spread, limiting any assessment of the impact of individual measures. There have been no interventional studies of non-pharmaceutical control measures to reduce COVID-19 infection in care homes, but a Cochrane rapid review (published in September 2021) identified 11 observational and 11 modelling studies, all from high-income countries.(5) The review grouped interventions into entry regulations (e.g. reducing visitors), contact regulating and transmission-reducing measures (e.g. Personal Protective Equipment, PPE), surveillance (symptomatic and asymptomatic testing), and outbreak control measures. Across these domains the quality of evidence was poor. In addition, there was widespread recognition that some of these measures, such as preventing visitors from entering the care home, were associated with significant harm.

Throughout the pandemic, testing has been used in three ways to reduce transmission of infection: 1) symptomatic testing, 2) testing during outbreaks to reduce their duration and severity and 3) regular, asymptomatic testing.

For peer review only - http://bmjopen.bmj.com/site/about/guidelines.xhtml

In the UK, compliance with regular testing may have been driven by national policies incentivising testing, including with financial support (e.g., Adult Social Care Rapid Testing Fund introduced in January 2021, Infection Control Fund introduced May 2020).(6,7) However, relatively few published studies have examined how these influence compliance with asymptomatic testing in care homes. We conducted a rapid systematic review, spanning January 2020 to July 2022. It highlighted 14 international papers,(8–21) published in English. No studies used an experimental design, and none reported, or evaluated, interventions designed to improve compliance with SARS-CoV-2 testing. The papers used a range of designs (e.g., qualitative, cross-sectional quantitative, consensus building). Together these studies highlight the *multi-levelled* factors that have shaped adherence with SARS-CoV-2 testing in care homes. We then used the behaviour change wheel (22) as an approach to develop systematically potentially useful intervention content from the factors influencing testing identified within the literature. Subsequently, through a series of stakeholder engagement events with diverse care home staff and representatives from the care home sector, we agreed the content of a multi-level intervention designed to maintain compliance with twice weekly lateral flow device (LFD) testing for COVID-19 within intervention care homes ('Test to Care').

There remains a lack of evidence on whether the benefits of regular testing for COVID-19 outweigh its harms, and if so, under which scenarios. There have been no attempts in the literature to consolidate the considerable expertise and learning on how to ensure compliance with testing in this setting. From a policy perspective, the key question remains over appropriate thresholds for turning testing 'on' and 'off' in response to varying levels of 'COVID-19 threat' (e.g., high/low levels of infection in the community; the emergence of novel COVID-19 variants).

We posit that the best approach to address these questions is through a randomised clinical trial. Randomisation overcomes the problem of substantial heterogeneity among care homes as they vary in resident population, care provision, and uptake of control measures such as vaccination in staff, or use of facemasks, which limit the conclusions that can be drawn from observational studies. Although there are significant challenges associated with undertaking a trial in care homes in a changing policy and epidemiological context, there is

For peer review only - http://bmjopen.bmj.com/site/about/guidelines.xhtml

an urgent need for high-quality evidence to inform the future use of testing for SARS-CoV-2 and potentially other infections in this setting.

Study aims

We will investigate whether continued use of regular asymptomatic testing in staff is a feasible, effective, and cost-effective strategy to reduce the impact of COVID-19 in care homes. Findings will inform testing policy across the United Kingdom (UK) for COVID-19 and add to knowledge on the use of testing in care homes to prevent other respiratory viruses, such as influenza. These objectives will be delivered through a series of five interlinked work-packages (WPs) which are described in detail in Supplementary File 1.

Methods and analysis

Study Design

VIVALDI-CT is a multi-centre, open label, cluster randomised controlled, phase III/IV superiority trial.

Each eligible care home will be randomised to either standard care (SARS-CoV-2 testing policy for care home staff that is in place nationally at the time of trial operation), or regular asymptomatic testing of care home staff for COVID-19 using Lateral Flow Devices (LFDs) combined with support payments for sickness absence and agency staff backfill.

Study Setting

VIVALDI-CT will take place in up to 280 residential and/or nursing homes in England providing care to adults aged >65 years.

Recruitment

Due to rapid timescales for trial delivery, and the need to streamline and centralise data collection we will primarily partner with providers that manage multiple care homes. We will first contact the senior management teams of providers that we have previously worked with in the Vivaldi study (23) to determine if they are interested in trial participation.

For peer review only - http://bmjopen.bmj.com/site/about/guidelines.xhtml

Providers will be asked to supply a list of eligible care homes and confirm that the care home manager has provided consent for each listed home to participate. If we are unable to recruit sufficient homes from the Vivaldi network, we will work with provider representative organisations (e.g., National Care Forum, Care England, National Care Association) to identify other eligible providers.

Homes will be selected to capture diversity in care home size, population (nursing / residential / dementia care), ethnicity, geographical location, rural/urban and provider type (for-profit / not-for-profit). Inclusive participation will be a focus by ensuring larger and smaller care groups are included in trial, as well as focusing on diverse settings.

Inclusion and Exclusion Criteria

Only care home staff are eligible to participate in the testing intervention. This includes temporary (agency) staff with no restrictions i.e., catering, administrative, and maintenance staff, in addition to those in a resident-facing role. However, all care home staff, as well as residents, visitors, and relatives, are eligible to participate in interviews undertaken as part of the trial's process evaluation. All care home residents at participating homes are eligible for data collection and analysis of the outcomes specified.

Visitors, residents, and relatives are not eligible to take part in the testing intervention. Staff who visit the care home to provide care but are not employed by the care home e.g., GPs, health visitors, are not eligible to take part in either the interviews or the testing intervention.

Primary outcome

The primary outcome is the incidence of COVID-19 related hospital admissions in residents defined as admissions with a relevant International Statistical Classification of Diseases and Related Health Problems 10th revision (ICD-10) code (COVID hospitalisations to be defined as any hospital admission record with a primary or secondary ICD10 code of 'U071') and/or admissions in residents who test positive for COVID-19 within 24 hours following admission or in the 7 days before hospital admission. This is considered the most important outcome for policymakers.

For peer review only - http://bmjopen.bmj.com/site/about/guidelines.xhtml

Secondary outcomes

Although we have adopted a healthcare/NHS perspective for the primary outcome, we recognise the importance of capturing outcomes that are relevant to the social care sector, such as outbreaks and care home closures. This is reflected in our choice of secondary outcomes:

- Incidence rate of hospital admissions (all-cause) in residents for non-elective care, measured as events per 100,000 person-days of follow-up over the duration of the trial.
- Incidence rate of COVID-associated mortality in residents, measured as events per 100,000 person-days of follow-up over the duration of the trial.
- Incidence of all-cause mortality in residents, measured as events per 100,000 person-days of follow-up over the duration of the trial.
- Testing uptake in staff, measured as proportion of staff at each home participating in testing during each week of the trial.
- Prevalence of SARS-CoV-2 among staff who test, measured as proportion of staff with positive test result among those with at least one test recorded during each week of the trial.
- Incidence rate of SARS-CoV-2 infections detected in residents, measured as events per 100,000 person-days of follow-up over the duration of the trial.
- Incidence rate of home-level outbreaks, measured as events per 1000 days of follow-up over the trial duration.
- Duration of outbreaks, measured as days from first to last case within outbreaks occurring within the trial period.
- Incidence rate of care home closures due to outbreaks, measured as events per 1000 days of follow-up over the duration of the trial.
- Proportion of staff per home who are off sick during each week of the trial.
- Proportion of all shifts filled by agency staff at each home each week.
- Costs per test.
- Testing metrics e.g., staff time taken to conduct the test at work.
- The impact of testing on resident, staff and visitors e.g., Social Care related Quality of Life collected via interviews in WP3.

For peer review only - http://bmjopen.bmj.com/site/about/guidelines.xhtml
For peer review only

Sample Size

Based on observational data from the VIVALDI study we found that over the 3-month period of January-March 2022 1.8% of residents had a COVID-19 related hospital admission, and the Intra-Cluster Correlation (ICC) across homes was 0.003 (95% CI 0.000-0.007). We assume that we will observe a cumulative incidence of around 3.0% in the trial, which would require a trial duration of 5-6 months if the incidence rate is similar to that in winter 2021/2022, in combination with a conservative ICC value of up to 0.01 (higher in line with the higher cumulative incidence compared to 3 months), and an average care home size of 35 residents with coefficient of variation in size of 0.5. With a total of 280 homes randomised 1:1 to trial arms and taking the usual two-sided test at 5% significance level, the design provides 84% power to detect a reduction in COVID-19 related admissions due to intervention to 1.9% (relative risk 0.63).

Timeline

The trial programme will run from November 2022 to April 2024. The recruitment of care home providers and operation of the intervention will take place between December 2022 and March 2023, with the possibility that this will be extended if deemed necessary for data collection. Supplementary File 2 shows a participant timeline and Figure 1 is a schematic of the trial pathway.

Intervention allocation

Care homes will be randomised on a 1:1 ratio. If all providers are ready for trial participation at the same time, then all participating homes will be randomised at the same time. Otherwise, the homes from different providers will be randomised in a phased approach, as they become ready. Randomisation will be performed by the trial statistician based on pseudo-random number generation after trial enrolment and before intervention implementation. Restricted randomisation (specifically covariate constrained randomisation) will be used to ensure balance on care home provider, size, and region.

For peer review only - http://bmjopen.bmj.com/site/about/guidelines.xhtml

Blinding

Researchers and staff of participating care homes will not be blinded to their intervention allocation, as this would not be feasible.

Data collection

To facilitate trial set-up and minimise the burden on care home staff, much of the data for analysis will be obtained from routinely collected healthcare information held within the UK COVID-19 Datastore. (24) This will include results of LFD and PCR tests for SARS-CoV-2 for staff and residents, information on hospital admissions and deaths for residents, and vaccination history for residents. Data within the COVID-19 Datastore are linked to a pseudonymised ID at the level of each individual, which can be linked to Care Quality Commission-ID (CQC-ID), a unique ID number provided by the CQC to identify each care home, for participating care homes and associated staff or resident status. A new study specific pseudonymised ID will be created for each individual before export of data to UCL, in order to prevent any theoretical possibility of reidentification.

Hospital admissions data are linked to ICD-10 diagnostic codes (including COVID-19 codes) however, there is a lag of several months in the assignment of these codes. To allow timely monitoring of data quality for the primary outcome and limit the risk of omitting or double-counting hospital admissions in residents, during the intervention period providers will be asked to upload weekly lists of COVID-associated hospital admissions in residents from participating care homes to the COVID-19 Datastore. Linkage to individual pseudonymised IDs will allow comparison to the routinely collected hospital admission data once available.

To inform estimates of incidence rates which form the primary and secondary outcomes, we will collect the total number of residents in the home on a weekly basis from providers as this will allow us to estimate the denominator. We will also collect the total number of staff on a weekly basis from each home to inform estimates of testing uptake and explore the feasibility of collecting data on the number of staff who opt-out of asymptomatic testing (in the intervention arm).

Care home level (aggregate) data will be collected from providers on dates of care home closures, use of disease control measures, staff sickness absence and employment of agency

For peer review only - http://bmjopen.bmj.com/site/about/guidelines.xhtml

staff to inform health economic analyses. We will explore the feasibility of collecting care home level data on fees paid by residents who are funded by the local authority, and whether it is feasible to collect more detailed information on healthcare utilisation, such as primary care consultations and use of antivirals in residents.

Data on outbreak events (dates, size) will be obtained from the United Kingdom Health Security Agency (UKHSA) Adult Social Care Team. Data on the local incidence of COVID-19 and co-circulation of other respiratory viruses will be obtained from the UKHSA and/or the Office of National Statistics (ONS) Covid Infection Survey.

Data management

Individual-level trial data will be stored in the UCL Data Safe Haven (25) which is hosted by UCL. All identifiable data will be held only by individual care homes or NHS England (NHSE), who will act as Data Processor on behalf of UCL. These databases are protected by multi-layer firewalls with full data encryption at rest and in transit.

For qualitative interviews, data collection will occur remotely using secure communication methods and be conducted by University of Strathclyde (UoS) researchers. Upon completion of transcription at UoS, the pseudo-anonymised data will be stored on the UoS network in a secure, restricted access folder for 5 years from the time of end of trial. Raw data will be destroyed once transcription and quality checks have been performed. Consent forms obtained via interviews and focus groups in the process evaluation will also be stored securely.

Statistical analysis

Analysis of the primary outcome, and secondary outcomes expressed as event incidence, will be based on Poisson or negative binomial regression with cluster-robust standard errors, adjusting for calendar time and key care home characteristics used in the restricted randomisation such as provider, region, and size. We will also explore whether the intervention effect differs according to care home size, and other characteristics such as proportion of temporary staff. Unadjusted effect estimates from these analyses will be reported for completeness. Using interaction terms, we will explore whether the effect of the intervention on the primary outcome differed between time periods defined by the

For peer review only - http://bmjopen.bmj.com/site/about/guidelines.xhtml

national recommendations for testing in the routine care arm, should these change during data collection.

Analysis of the primary outcome will include all trial care homes (intention to treat analysis), and so represent a treatment policy estimand. We will also define an implementation score based on the frequency and proportion of staff testing at each home based on data the homes provide, which may vary over time. As an exploratory analysis we will assess whether the primary outcome is associated with this implementation score within the intervention arm and express the effect of the intervention relative to control arm for different levels of implementation. This analysis will be based on the same regression method as used for the primary analysis.

Health Economic Analysis

The health economic analysis will investigate the cost-effectiveness and cost-consequences of the testing intervention taking a NHS, Personal Social Services, and a societal perspective using a lifetime horizon (according to care home resident average age and life expectancy). The within-trial costs and outcomes in intervention and control groups will be examined from each perspective. Cost-effectiveness of the intervention in terms of the primary outcome and in terms of all-cause mortality will also be examined. Costs of admission will be excluded from the total costs under consideration in this case.

We will also examine cost-effectiveness in terms of the secondary outcomes of cases prevented and resident deaths prevented, and outcomes of hospital admission and number of outbreaks alongside costs offset/additional costs incurred in a cost-consequences analysis.

Process Evaluation

There is major diversity across care homes, for example in terms of provision of care, resident population, care home size, and the care home workforce. As a result, it is essential to consider the feasibility and sustainability of the intervention and how contextual factors might impact on the ability to scale it, if the trial suggests it is effective and cost-effective. These issues will be addressed in the process evaluation which aims to

For peer review only - http://bmjopen.bmj.com/site/about/guidelines.xhtml

understand intervention roll out and identify areas for optimisation to inform future intervention scale-up, should the testing approach prove effective and cost effective.

The objectives of the process evaluation are:

- To determine intervention acceptability.
- To determine the role of context in shaping the way, the intervention operated.
- To determine what can be learned about intervention fidelity and adaptation.
- To determine which intervention components worked as anticipated and which need further modification.
- To investigate unanticipated intervention effects.
- To determine what can be learned from the control group.

The process evaluation will develop implementation guidance and training packages ready for future scale up as well as details of minimal care home requirements and staff competencies necessary for intervention delivery.

Qualitative data will be collected from 28 (10%) care homes evenly distributed across each intervention and control arms and spaced across time.

Patient and Public Involvement

Patient public involvement (PPI) has already informed the development of this programme, by highlighting the barriers to testing and the need to capture its adverse impacts on staff, residents, and providers. Public advisors have also emphasised the importance of developing a strong plan for implementation, recognising the financial implications of long-term use of testing and sickness payments, informing our emphasis on implementation in WP5.

The PPI team will deliver the following objectives:

- To ensure that the ‘voice and views’ of the public regarding regular testing for COVID-19 are heard by the research team and the wider stakeholder group.
- To create an open, inclusive culture enabling effective communication between the study team, PPI group and the wider stakeholder and oversight groups.

For peer review only - http://bmjopen.bmj.com/site/about/guidelines.xhtml

- To agree an approach to communicate outputs from the trial to different audiences, including care home staff, residents and their families and the public using a variety of media.

Ethics and dissemination

Study monitoring

An independent Trial Steering Committee (TSC) and Data Monitoring and Ethics Committee (DMEC) will be formally responsible for programme oversight, ensuring the study is conducted in compliance with regulations. The DMEC will also be responsible for monitoring the accumulating data and making recommendations to the TSC on whether the trial should continue as planned.

A Trial Management Group (TMG) will be responsible for the design, coordination, and strategic management of the trial.

Safety reporting

Staff at the sites randomised to asymptomatic testing will report the occurrence of Serious Adverse Events (SAEs) considered 'related' to the intervention only. In such cases site personnel will complete a Serious Adverse Event (SAE) report within 24 hours of notification of the event. Clinical review of any SAEs will take place and be reported to the Research Ethics Committee if deemed both 'related' to the trial intervention and 'unexpected' in line with UK Health Research Authority non-drug trial reporting requirements.

Research ethics approval

The study has been approved by the London - Bromley Research Ethics Committee (reference number 22/LO/0846) and the Health Research Authority (HRA) (22/CAG/0165).

Consent and opt out

Care home providers and home managers will be asked if their care home(s) are willing to participate in the trial. High staff turnover, in conjunction with the large number of care

For peer review only - http://bmjopen.bmj.com/site/about/guidelines.xhtml

homes participating in the trial, means that it is not feasible to obtain individual consent from staff or regarding the use of testing data. Staff and residents have the option of opting out from the processing and analysis of their individual-level data within this study at any time during the study.

Identifiable data submitted by care homes as part of the study will be pseudonymised by NHS England before it is provided to the research team. This study has section 251 support to allow the disclosure of confidential patient information (regarding testing in staff) from care homes to NHS England, for the purposes of monitoring uptake of the testing in the control and intervention arms of the trial.

The study will also collect limited individual-level identifiable data from residents to ensure the primary outcome can be determined accurately. It is not feasible to seek individual-level consent from every resident for the use of these data due to high levels of cognitive impairment in residents and excluding data from a large proportion of residents would compromise the scientific value of the trial and the subsequent generalisability of trial findings. This study has section 251 support to allow the disclosure of confidential patient information (regarding residents admitted to hospital) from care homes to NHS England, for the purposes of linkage to the COVID-19 datastore and to enable NHS England to use confidential patient information from SARS-CoV-2 tests to link to other NHS datasets within the COVID-19 datastore.

Care home managers in the subset of homes selected for qualitative data collection (focus groups or one to one interviews) will be asked to disseminate recruitment materials to staff within the home via word of mouth, email, or other routine modes of communication. Upon receipt of staff contact details, interested staff will then be sent participant information sheets about the study, given the option to ask questions about the study, complete on-line consent forms and provide brief sociodemographic details to enable the study team to monitor total sample composition. Having checked on-line consent has already been given and after exploring any remaining unanswered questions raised by the PIS, the participants will be asked to also give recorded oral consent to participate.

For peer review only

For peer review only - http://bmjopen.bmj.com/site/about/guidelines.xhtml

Confidentiality

All data will be handled in accordance with the Data Protection Act 2018, the UK General Data Protection Regulation (UK GDPR) and subsequent updates and amendments.

Dissemination policy

The results of the trial will be disseminated regardless of the direction of effect. The publication of the results will comply with a trial-specific publication policy and will include submission to open access journals.

A lay summary of the results will also be produced to disseminate the results to participants. A summary of results will be included online in the publicly accessible Health Research Authority (HRA) website within 12 months of date of trial closure. A Statistical Analysis Plan will also be published under open access arrangements.

Trial Registration and Reporting Guidelines

The VIVALDI-CT was registered with the International Standard Randomised Controlled Trial Number website (ISRCTN 13296529) (26) on 5 December 2022 and the protocol adheres to the SPIRIT 2013 Statement (27).

Author Statement

NA, JB, MKr, LS and PF developed the study protocol and contributed to the writing of the manuscript. OS and AC were involved in development of the statistical analysis facets of the trial and contributed to the writing of the manuscript. CH and MKn were involved in development of the health economic analysis facets of the study and contributed to the writing of the manuscript. RL was involved in development of the process evaluation of the study and contributed to the writing of the manuscript. DC and JC were involved in development of the health-related quality of life facets of the study and contributed to the writing of the manuscript. LG was involved in development of the economic modelling of the study and contributed to the writing of the manuscript. ICS is the trial manager for the study. RF will oversee data collection and management. MR, AV, AG, NF and SH are co-applicants responsible for supporting the operationalisation of the study. LS and PF are the

For peer review only - http://bmjopen.bmj.com/site/about/guidelines.xhtml

co- chief investigators for the study. All authors critically reviewed and approved the final version.

Funding

This work is supported by the NIHR Health and Social Care Delivery Research (HSDR) Programme number [154310]. Costs associated with SARS-CoV-2 testing including support payments for care home staff and for care homes to fund agency staff backfill will be funded by the UK Health Security Agency (UKHSA).

Sponsor

The VIVALDI-CT is sponsored by University College London, represented by the UCL Comprehensive Clinical Trials Unit (UCL CCTU).

Competing interests

None declared.

Study Status

Current Protocol – Version 3.0 dated 04 Jan 2023. At the time of protocol submission, recruitment of sites had been completed.

Data Statement

No data are associated with this article.

Data from the trial will be available for sharing between the trial researchers as detailed in the Data Sharing Agreement between the institutions hosting the VIVALDI-CT researchers.

Requests for access to trial data will be considered, and approved in writing where appropriate, after formal application to the TSC. Considerations for approving access are documented in the TSC Terms of Reference.

For peer review only

For peer review only - http://bmjopen.bmj.com/site/about/guidelines.xhtml

Acknowledgements

We are grateful to the care homes who have already agreed to participate in the VIVALDI-CT. We would also like to acknowledge the role of the care homes participating in the VIVALDI Study for sharing their learning from the pandemic with us.

We also acknowledge the support of the independent members of the Trial Steering Committee (TSC):

- Prof Alistair Hay (University of Bristol)
- Dr Jennifer Thompson (LSHTM)
- Dr Michael Larkin (Aston University)
- Zoe Fry (Outstanding Society)
- Samantha Crawley (Bracebridge)
- Margaret Ogden (independent PPI member)

As well as the Data Monitoring and Ethics Committee (DMEC):

- Prof Karla Hemming (University of Birmingham)
- Dr Tania Kalsi (Guy's and St Thomas' NHS Foundation Trust)
- Dr Terry Quinn (University of Glasgow)

We would also like to thank members of the UKHSA:

- Dr Tom Fowler (UKHSA)
- Alex Barton (DHSC)
- Prof Jackie Cassell (UKHSA)
- Dr Sarah Tunkel (UKHSA)

For peer review only

For peer review only - http://bmjopen.bmj.com/site/about/guidelines.xhtml

Figures and tables

Supplementary file 1: Description of work packages in the VIVALDI-CI

Supplementary file 2: Participant timeline

For peer review only

For peer review only - http://bmjopen.bmj.com/site/about/guidelines.xhtml

Figure Legend

Figure 1: Flow diagram of trial pathway

For peer review only

For peer review only - http://bmjopen.bmj.com/site/about/guidelines.xhtml

References

1. Kinley J, Hockley J, Stone L, et al. The provision of care for residents dying in UK nursing care homes. *Age Ageing*. 2014;43(3):375-379. doi:10.1093/ageing/aft158

2. Alzheimer's Society. *Dementia UK Update*.; 2014. Accessed November 27, 2021. https://www.alzheimers.org.uk/sites/default/files/migrate/downloads/dementia_uk_update.pdf

3. Levin AT, Jylhävä J, Religa D, Shallcross L. COVID-19 prevalence and mortality in longer-term care facilities. *Eur J Epidemiol*. 2022;37(3):227-234. doi:10.1007/s10654-022-00861-w

4. Kaelen S, van den Boogaard W, Pellecchia U, et al. How to bring residents' psychosocial well-being to the heart of the fight against Covid-19 in Belgian nursing homes—A qualitative study. Kotozaki Y, ed. *PLOS ONE*. 2021;16(3):e0249098. doi:10.1371/journal.pone.0249098

5. Stratil JM, Biallas RL, Burns J, et al. Non-pharmacological measures implemented in the setting of long-term care facilities to prevent SARS-CoV-2 infections and their consequences: a rapid review. Cochrane Public Health Group, ed. *Cochrane Database Syst Rev*. 2021;2021(9). doi:10.1002/14651858.CD015085.pub2

6. Adult Social Care Rapid Testing Fund. GOV.UK. Accessed October 7, 2022. https://www.gov.uk/government/publications/adult-social-care-rapid-testing-fund

7. Adult Social Care Infection Control Fund. GOV.UK. Accessed July 26, 2022. https://www.gov.uk/government/publications/adult-social-care-infection-control-fund

8. Gray KL, Birtles H, Reichelt K, James IA. The experiences of care home staff during the COVID-19 pandemic: A systematic review. *Aging Ment Health*. 2022;26(10):2080-2089. doi:10.1080/13607863.2021.2013433

9. Pétré B, Paridans M, Gillain N, et al. Factors influencing the adoption and participation rate of nursing homes staff in a saliva testing screening programme for COVID-19. *PloS One*. Published online June 30, 2022. doi:10.1371/journal.pone.0270551

10. Pétré B, Paridans M, Gillain N, et al. Acceptability of Community Saliva Testing in Controlling the COVID-19 Pandemic: Lessons Learned from Two Case Studies in Nursing Homes and Schools. *Patient Prefer Adherence*. 2022;16:625-631. doi:10.2147/PPA.S349742

11. Walker, J. Implementing COVID-19 infection prevention and control measures in long-term care settings. *Nurs Older People*. Published online April 5, 2022. doi:10.7748/nop.2022.e1384

12. Nyashanu M, Pfende F, Ms E. Triggers of mental health problems among frontline healthcare workers during the COVID-19 pandemic in private care homes and

For peer review only - http://bmjopen.bmj.com/site/about/guidelines.xhtml

domiciliary care agencies: Lived experiences of care workers in the Midlands region, UK. *Health Soc Care Community*. Published online February 2022. doi:10.1111/hsc.13204

13. Tulloch J, Micocci M, Buckle P, et al. Enhanced lateral flow testing strategies in care homes are associated with poor adherence and were insufficient to prevent COVID-19 outbreaks: results from a mixed methods implementation study. *Age Ageing*. Published online November 10, 2021. doi:10.1093/ageing/afab162

14. Kierkegaard P, Micocci M, McLister A, et al. Implementing lateral flow devices in long-term care facilities: experiences from the Liverpool COVID-19 community testing pilot in care homes- a qualitative study. *BMC Health Serv Res*. Published online October 25, 2021. doi:10.1186/s12913-021-07191-9

15. Oldfield B, DeCosta S, Petterson L, Lagarde S, Olson D. A Blueprint for Community Health Center and Nursing Home Partnership: Testing for COVID-19 among Residents and Staff at Long-term Care Facilities. *J Health Care Poor Underserved*. Published online 2021. doi:10.1353/hpu.2021.0001

16. Micocci M, Gordon A, Allen A, et al. COVID-19 testing in English care homes and implications for staff and residents. *Age Ageing*. Published online May 5, 2021. doi:10.1093/ageing/afab015

17. White E, Wetle T, Reddy A, Baier R. Front-line Nursing Home Staff Experiences During the COVID-19 Pandemic. *J Am Med Dir Assoc*. Published online January 2021. doi:10.1016/j.jamda.2020.11.022

18. Ouslander J, Grabowski D. COVID-19 in Nursing Homes: Calming the Perfect Storm. *J Am Geriatr Soc*. Published online October 2020. doi:10.1111/jgs.16784

19. Wasserman M, Ouslander J, Lam A, et al. Editorial: Diagnostic Testing for SARS-Coronavirus-2 in the Nursing Facility: Recommendations of a Delphi Panel of Long-Term Care Clinicians. *J Nutr Health Aging*. Published online 2020. doi:10.1007/s12603-020-1401-9

20. Gordon A, Goodman C, Achterberg W, et al. Commentary: COVID in care homes-challenges and dilemmas in healthcare delivery. *Age Ageing*. Published online August 24, 2020. doi:10.1093/ageing/afaa113

21. COVID-19 and policies for care homes in the first wave of the pandemic in European welfare states: Too little, too late? - Mary Daly, Margarita León, Birgit Pfau-Effinger, Costanzo Ranci, Tine Rostgaard, 2022. Accessed July 26, 2022. https://journals.sagepub.com/doi/full/10.1177/09589287211055672

22. Michie S, van Stralen MM, West R. The behaviour change wheel: A new method for characterising and designing behaviour change interventions. *Implement Sci*. 2011;6(1):42. doi:10.1186/1748-5908-6-42

For peer review only

For peer review only - http://bmjopen.bmj.com/site/about/guidelines.xhtml

23. Krutikov M, Palmer T, Donaldson A, et al. Study Protocol: Understanding SARS-Cov-2 infection, immunity and its duration in care home residents and staff in England (VIVALDI). *Wellcome Open Res*. 2021;5:232. doi:10.12688/wellcomeopenres.16193.2

24. NHS England. NHS COVID-19 Data Store. Accessed October, 7 2022. https://www.england.nhs.uk/contact-us/privacy-notice/how-we-use-your-information/covid-19-response/nhs-covid-19-data-store/

25. UCL. Data Safe Haven (DSH). Information Services Division. Published February 14, 2018. Accessed October 7, 2022. https://www.ucl.ac.uk/isd/services/file-storage-sharing/data-safe-haven-dsh

26. Shallcross L, Shallcross L, Enquiries C. Regular asymptomatic COVID-19 testing in care home staff. doi:10.1186/ISRCTN13296529

27. Chan AW, Tetzlaff JM, Altman DG, et al. SPIRIT 2013 statement: defining standard protocol items for clinical trials. *Ann Intern Med*. 2013;158(3):200-207. doi:10.7326/0003-4819-158-3-201302050-00583

For peer review only

For peer review only - http://bmjopen.bmj.com/site/about/guidelines.xhtml

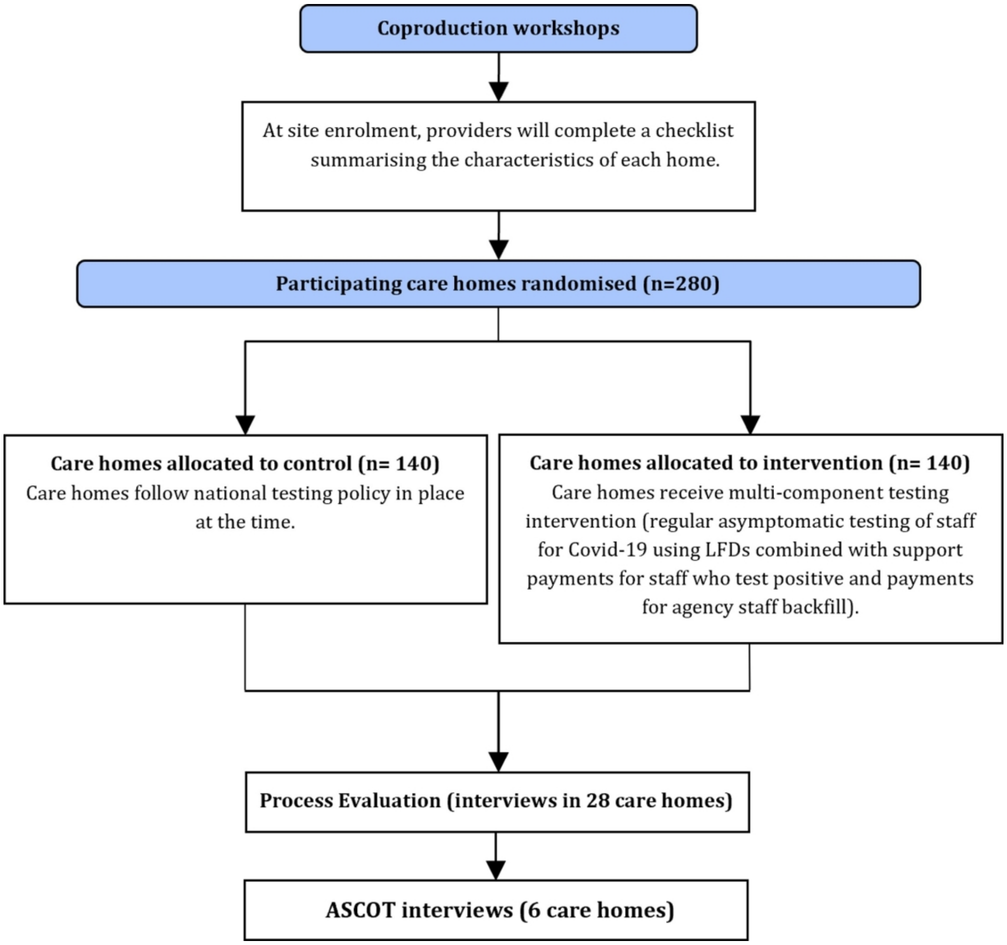

Figure 1: Flow diagram of trial pathway

166x156mm (330 x 330 DPI)

Supplementary File 1: Description of work packages in the VIVALDI-CT

Work Package	Aims	Participants	Methods	Data collection	Data analysis	Outcomes
WP1	To coproduce a sustainable testing intervention for care home staff.	Care home staff, providers and policymakers.	A series of workshops with care home stakeholders (e.g., home managers, staff, providers) and policymakers (including those with knowledge of testing logistics).<br><br>A workshop will also take place with residents with capacity to consent, relatives and visitors.	1) Consolidation of existing insights into routine testing gleaned through past experiences with the COVID-19 pandemic; 2) discussion of the intervention prototype; and 3) Operationalisation of intervention in ways which are likely to be acceptable and appropriate within the sector.	Data (transcript, detailed notes, images) will be analysed thematically to further develop the initial programme theory specifying important elements of the context, key intervention components, their mechanisms, and their relation to a hierarchy of outcome measures (e.g., health, social and financial outcomes).	Development of a testing intervention for use in WP2
WP2	To evaluate the effectiveness of the testing intervention compared to the recommended testing protocol that is in place at the time in a pragmatic cluster randomised trial.	Care home staff.	A cluster randomised controlled trial in which asymptomatic care home staff will be randomised at provider level to either test twice weekly using LFDs or to follow current national testing guidance.	At enrolment, providers will complete a checklist summarising characteristics of each participating care home. Each week during the testing period care home managers will be asked to record: 1) dates of hospital admissions for residents and their corresponding NHS	Analysis of the primary outcome, and secondary outcomes expressed as event incidence, will be based on Poisson or negative binomial regression with cluster-robust standard errors, adjusting for calendar time and key care home characteristics used in the restricted	The primary outcome will be the incidence of COVID-19 related hospital residents.

				numbers and/or identifiers, 2) number of staff and residents in each care home. The study will collect limited individual-level identifiable data from residents to ensure that the primary outcome of hospital admissions is accurate. Linkage to other routine datasets (hospitalisations, cause of death, COVID-19 test results, vaccination status).	randomisation such as provider, region, and size.	
WP3A	To understand the intervention roll out and identify areas for optimisation to inform future intervention scale-up, should the testing approach prove effective and cost effective	Care home staff	The intervention programme theory and associated logic models from WP1 will be used to undertake a parallel mixed methods process evaluation.<br>Interviews and focus groups will be undertaken with staff.	Data collection will be facilitated though virtual platforms (according to participant norms).<br><br>Quantitative data will include descriptive statistics from across all trial sites complemented by multivariate analyses of data sets where appropriate.	A combination of deductive and inductive thematic analysis will be used.<br><br>Interview/focus group transcriptions and qualitative survey data will be imported into NVivo10 software to facilitate data handling, organisation and coding. Analysis will firstly be	Implementation guidance and training packages ready for future scale up;<br><br>Details of minimal care home requirements and staff competencies necessary for

				Qualitative data will be collected from 28 (10%) care homes evenly distributed across each intervention and control arms and spaced across time. Selection criteria will focus on geography, socio-economic status of area, highest/lowest rates of infection. Heterogeneous samples of staff will be recruited. After consent is attained qualitative data will be collected primarily using focus groups. One to one interviews will also be possible if requested.	thematic and use a combination of deductive and inductive thematic analysis.	intervention delivery.
WP3B	To conduct a mixed methods, process evaluation and exploratory analysis to explore the impact of testing and outbreaks on social care related quality of life in home care residents	Care home residents	The Adult Social Care Outcomes Toolkit (ASCOT) will be used to assess the impact of the intervention and outbreaks on residents. ASCOT is a well-established tool for measuring social care related quality of life	In person interviews. If in-person resident interviews are not possible, Essential Caregivers (designated visitor allowed even during outbreaks) or members of staff will be interviewed.	The ASCOT tool will be used to assess SCRQoL at baseline, during/immediately after an outbreak or mid study, and at end of the study. ASCOT data will be combined with individual level demographic and health and aggregate	Outputs will also be integrated with findings from WP3A to provide a holistic assessment of the acceptability and feasibility of the testing intervention.

For peer review only

			(SCRQoL). We will establish an ASCOT cohort of six care homes stratified by type and size, distributed equally across control and intervention arms. Homes taking part in the qualitative interviews in WP3A are excluded.		home level data to give a comprehensive description of SCRQoL.	
WP4A	To evaluate the costs and benefits of the testing intervention.	NHS, providers, residents, families and staff.	We will examine within-trial costs and outcomes in intervention and control groups from each perspective. We will examine the cost-effectiveness of the intervention in terms of the primary outcome and in terms of all-cause mortality. The costs of admission will be excluded from the total costs under consideration in this case. We will also examine cost-effectiveness in terms of the secondary outcomes	We will examine the cost-effectiveness of the intervention in terms of the primary outcome, excluding cost of admission from total cost. We will also examine cost-effectiveness in terms of secondary outcomes of cases prevented and resident deaths prevented. We will examine outcomes of hospital admission and number of outbreaks alongside costs offset/additional costs	Generalised linear models appropriate to counts (hospital admissions, numbers of outbreaks, cases) or binary outcomes (deaths) and costs will be applied. For the purposes of the cost-effectiveness analyses, these will take into account possible correlations between costs and outcomes either by non-parametric bootstrapping of separate regressions or joint modelling approaches such as seemingly unrelated	Cost-effectiveness of the intervention in terms of the primary trial outcome (incidence of COVID-19 related hospital admission in residents.)<br><br>Cost-effectiveness in terms of the secondary outcomes of cases prevented and resident

For peer review only

			of cases prevented and resident deaths prevented. We will examine the outcomes of hospital admission and number of outbreaks alongside costs offset/additional costs incurred in a cost-consequences analysis.	incurred in a cost-consequences analysis. investigate the cost-effectiveness and cost-consequences of the testing intervention taking both a NHS, Personal Social Services, and a societal perspective.	regressions. Where individual level data are available, analyses will take a multilevel approach to adjust for clustering at the care home level either by two-stage bootstrapping of separate regressions or simultaneous modelling. Incremental cost-effectiveness ratios will be presented, net benefit calculated over a range of willingness to pay values for gains in outcomes to generate cost-effectiveness acceptability curves.	deaths prevented.<br><br>Cost-consequences of hospital admission and number of outbreaks alongside costs offset/additional costs incurred.
WP4B	To model costs and benefits of the testing intervention under different scenarios	NHS, providers, residents, families and staff.	A compartmental model will be built to study transmission of infection and infer the proportion of COVID-19 infections and deaths averted by the intervention under different epidemiological	Data from WP4A will be used.	The model will consider two populations i.e., home residents and staff, and take into account both symptomatic and asymptomatic cases, as well as hospital admissions and deaths.	Estimate of the projected cost-effectiveness of the intervention.

For peer review only

			scenarios (e.g., high/low community incidence of infection, care home population size etc).<br><br>Two scenarios will be modelled: 1) standard care: residents and staff get tested only if they show symptoms; and 2) intervention: standard care plus regular testing of all staff for COVID-19.		The model will be calibrated to trial results and modelling results will be projected in time by extending the time horizon. Unit costs calculated in the first part of the economic analysis will be discounted to future years values and associated to modelling results to estimate the projected cost-effectiveness of the intervention.<br><br>We will also explore the short and long-term costs of the testing intervention under different epidemiological scenarios (e.g., high/low community incidence of infection).	
WP5	To coproduce recommendations on the use of	Providers, care home staff, the NHS and	Three round-table discussions for a	Discussions will be based on world café methodological	Data (transcript, detailed notes, images) will be analysed thematically.	Formal mechanism to ensure

For peer review only - http://bmjopen.bmj.com/site/about/guidelines.xhtml

	regular testing for policymakers	primary care, policymakers and public health teams at national and regional / local level, community, residents and families.	maximum of 20 stakeholders.	principles. The views of the community, residents and families will be represented through organisations such as Healthwatch, the Residents and Relatives association and the VIVALDI-CT PPI group.		stakeholders are aware and involved in the work as it progresses and to enable the sector to prepare and plan for results and their implications.<br><br>Production of recommendations on the use of regular testing for policymakers.

For peer review only

Supplementary File 2: Participant Timeline

**Trial visit number**	**Baseline**	**Care home data collection period**							
**Month**	**Month 0**	**Months 1-4**		**Survey**	**Routine COVID-19 datastore**	**Uploaded to Foundry by provider data manager**	**To be reported as and when occurs**	**Weekly collection from providers**	**Participants**
		**Sites on Intervention arm**	**Sites on Standard Care arm**						
Demography	x			x	x				
Resident registry (patient type)	x			x					
Vaccination status	x				x				
Care home characteristics	x			x	x				
Number of residents per home (weekly)		x	x					x	
COVID-associated hospital admission events in residents		x	x		x	x			
Number of staff absent from work		x	x					x	
Total shifts at home this week (sum for all staff)		x	x					x	
Number of agency shifts		X	x					x	

For peer review only

Number of staff per home (weekly)		x	x					x	
Number of staff opting out of asymptomatic testing (weekly)*		x						x	
Routine data on LFD and PCR tests (staff+residents)		x	x		x				
Routine data on hospital admissions (residents)		x	x		x				
Routine data on mortality (residents)		x	x		x				
Outbreak event data		x	x					x	
Safety reporting		x	x				x	x	
Process Evaluation (3a)									
Focus groups		x	x						x
Interviews		x	x						x

* If the control arm starts routine testing, then collected from both arms

For peer review only

SPIRIT
STANDARD PROTOCOL ITEMS: RECOMMENDATIONS FOR INTERVENTIONAL TRIALS

SPIRIT 2013 Checklist: Recommended items to address in a clinical trial protocol and related documents*

Section/item	Item No	Description	Page No
**Administrative information**			
Title	1	Descriptive title identifying the study design, population, interventions, and, if applicable, trial acronym	1
Trial registration	2a	Trial identifier and registry name. If not yet registered, name of intended registry	16
	2b	All items from the World Health Organization Trial Registration Data Set	1-17
Protocol version	3	Date and version identifier	17
Funding	4	Sources and types of financial, material, and other support	16
Roles and responsibilitie s	5a	Names, affiliations, and roles of protocol contributors	16-18
	5b	Name and contact information for the trial sponsor	16
	5c	Role of study sponsor and funders, if any, in study design; collection, management, analysis, and interpretation of data; writing of the report; and the decision to submit the report for publication, including whether they will have ultimate authority over any of these activities	16,17
	5d	Composition, roles, and responsibilities of the coordinating centre, steering committee, endpoint adjudication committee, data management team, and other individuals or groups overseeing the trial, if applicable (see Item 21a for data monitoring committee)	16-18
**Introduction**			
Background and rationale	6a	Description of research question and justification for undertaking the trial, including summary of relevant studies (published and unpublished) examining benefits and harms for each intervention	4-5
	6b	Explanation for choice of comparators	4-5
Objectives	7	Specific objectives or hypotheses	5-6

For peer review only

Trial design	8	Description of trial design including type of trial (eg, parallel group, crossover, factorial, single group), allocation ratio, and framework (eg, superiority, equivalence, noninferiority, exploratory)	6

Methods: Participants, interventions, and outcomes

Study setting	9	Description of study settings (eg, community clinic, academic hospital) and list of countries where data will be collected. Reference to where list of study sites can be obtained	6
Eligibility criteria	10	Inclusion and exclusion criteria for participants. If applicable, eligibility criteria for study centres and individuals who will perform the interventions (eg, surgeons, psychotherapists)	7
Interventions	11a	Interventions for each group with sufficient detail to allow replication, including how and when they will be administered	9
	11b	Criteria for discontinuing or modifying allocated interventions for a given trial participant (eg, drug dose change in response to harms, participant request, or improving/worsening disease)	13
	11c	Strategies to improve adherence to intervention protocols, and any procedures for monitoring adherence (eg, drug tablet return, laboratory tests)	6
	11d	Relevant concomitant care and interventions that are permitted or prohibited during the trial	6
Outcomes	12	Primary, secondary, and other outcomes, including the specific measurement variable (eg, systolic blood pressure), analysis metric (eg, change from baseline, final value, time to event), method of aggregation (eg, median, proportion), and time point for each outcome. Explanation of the clinical relevance of chosen efficacy and harm outcomes is strongly recommended	7-8
Participant timeline	13	Time schedule of enrolment, interventions (including any run-ins and washouts), assessments, and visits for participants. A schematic diagram is highly recommended (see Figure)	9, Table 2
Sample size	14	Estimated number of participants needed to achieve study objectives and how it was determined, including clinical and statistical assumptions supporting any sample size calculations	9
Recruitment	15	Strategies for achieving adequate participant enrolment to reach target sample size	6

Methods: Assignment of interventions (for controlled trials)

Allocation:

For peer review only - http://bmjopen.bmj.com/site/about/guidelines.xhtml

Sequence generation	16a	Method of generating the allocation sequence (eg, computer-generated random numbers), and list of any factors for stratification. To reduce predictability of a random sequence, details of any planned restriction (eg, blocking) should be provided in a separate document that is unavailable to those who enrol participants or assign interventions	9
Allocation concealment mechanism	16b	Mechanism of implementing the allocation sequence (eg, central telephone; sequentially numbered, opaque, sealed envelopes), describing any steps to conceal the sequence until interventions are assigned	9
Implementation	16c	Who will generate the allocation sequence, who will enrol participants, and who will assign participants to interventions	9
Blinding (masking)	17a	Who will be blinded after assignment to interventions (eg, trial participants, care providers, outcome assessors, data analysts), and how	10
	17b	If blinded, circumstances under which unblinding is permissible, and procedure for revealing a participant's allocated intervention during the trial	N/A
**Methods: Data collection, management, and analysis**			
Data collection methods	18a	Plans for assessment and collection of outcome, baseline, and other trial data, including any related processes to promote data quality (eg, duplicate measurements, training of assessors) and a description of study instruments (eg, questionnaires, laboratory tests) along with their reliability and validity, if known. Reference to where data collection forms can be found, if not in the protocol	10-11
	18b	Plans to promote participant retention and complete follow-up, including list of any outcome data to be collected for participants who discontinue or deviate from intervention protocols	10-11
Data management	19	Plans for data entry, coding, security, and storage, including any related processes to promote data quality (eg, double data entry; range checks for data values). Reference to where details of data management procedures can be found, if not in the protocol	11
Statistical methods	20a	Statistical methods for analysing primary and secondary outcomes. Reference to where other details of the statistical analysis plan can be found, if not in the protocol	11-12
	20b	Methods for any additional analyses (eg, subgroup and adjusted analyses)	11-12
	20c	Definition of analysis population relating to protocol non-adherence (eg, as randomised analysis), and any statistical methods to handle missing data (eg, multiple imputation)	10-11

Methods: Monitoring

Data monitoring	21a	Composition of data monitoring committee (DMC); summary of its role and reporting structure; statement of whether it is independent from the sponsor and competing interests; and reference to where further details about its charter can be found, if not in the protocol. Alternatively, an explanation of why a DMC is not needed	13
	21b	Description of any interim analyses and stopping guidelines, including who will have access to these interim results and make the final decision to terminate the trial	13
Harms	22	Plans for collecting, assessing, reporting, and managing solicited and spontaneously reported adverse events and other unintended effects of trial interventions or trial conduct	13
Auditing	23	Frequency and procedures for auditing trial conduct, if any, and whether the process will be independent from investigators and the sponsor	13

Ethics and dissemination

Research ethics approval	24	Plans for seeking research ethics committee/institutional review board (REC/IRB) approval	14
Protocol amendments	25	Plans for communicating important protocol modifications (eg, changes to eligibility criteria, outcomes, analyses) to relevant parties (eg, investigators, REC/IRBs, trial participants, trial registries, journals, regulators)	16
Consent or assent	26a	Who will obtain informed consent or assent from potential trial participants or authorised surrogates, and how (see Item 32)	14-15
	26b	Additional consent provisions for collection and use of participant data and biological specimens in ancillary studies, if applicable	14-15
Confidentialit y	27	How personal information about potential and enrolled participants will be collected, shared, and maintained in order to protect confidentiality before, during, and after the trial	15
Declaration of interests	28	Financial and other competing interests for principal investigators for the overall trial and each study site	17
Access to data	29	Statement of who will have access to the final trial dataset, and disclosure of contractual agreements that limit such access for investigators	17
Ancillary and post-trial care	30	Provisions, if any, for ancillary and post-trial care, and for compensation to those who suffer harm from trial participation	N/A

For peer review only - http://bmjopen.bmj.com/site/about/guidelines.xhtml

Dissemination policy	31a	Plans for investigators and sponsor to communicate trial results to participants, healthcare professionals, the public, and other relevant groups (eg, via publication, reporting in results databases, or other data sharing arrangements), including any publication restrictions	16
	31b	Authorship eligibility guidelines and any intended use of professional writers	16
	31c	Plans, if any, for granting public access to the full protocol, participant-level dataset, and statistical code	17
**Appendices**			
Informed consent materials	32	Model consent form and other related documentation given to participants and authorised surrogates	N/A
Biological specimens	33	Plans for collection, laboratory evaluation, and storage of biological specimens for genetic or molecular analysis in the current trial and for future use in ancillary studies, if applicable	N/A

*It is strongly recommended that this checklist be read in conjunction with the SPIRIT 2013 Explanation & Elaboration for important clarification on the items. Amendments to the protocol should be tracked and dated. The SPIRIT checklist is copyrighted by the SPIRIT Group under the Creative Commons "Attribution-NonCommercial-NoDerivs 3.0 Unported" license.

For peer review only

BMJ Open

Shaping care home COVID-19 testing policy: A Protocol for a pragmatic cluster randomised controlled trial of asymptomatic testing compared to standard care in care home staff (VIVALDI-CT).

Journal:	*BMJ Open*
Manuscript ID	bmjopen-2023-076210.R2
Article Type:	Protocol
Date Submitted by the Author:	23-Oct-2023

Complete List of Authors:	Adams, Natalie; Royal Free London NHS Foundation Trust; University College London, Institute of Health informatics<br>Stirrup , Oliver ; University College London, Institute for Global Health<br>Blackstone, James; University College London, Comprehensive Clinical Trials Unit<br>Krutikov, Maria; University College London, Institute of Health informatics<br>Cassell, Jackie; Brighton and Sussex Medical School, Department of Primary Care and Public Health; UK Health Security Agency<br>Cadar, Dorina; Brighton and Sussex Medical School, Department of Primary Care and Public Health; Brighton and Sussex Medical School, Centre for Dementia Studies, Department of Neuroscience<br>Henderson, Catherine; The London School of Economics and Political Science, Care Policy and Evaluation Centre<br>Knapp, Martin; The London School of Economics and Political Science, Care Policy and Evaluation Centre<br>Goscé, Lara; University College London, Institute for Global Health; London School of Hygiene & Tropical Medicine, Department of Infectious Disease Epidemiology<br>Leiser, Ruth; University of Strathclyde, Department of Psychological Sciences and Health<br>Regan, Martyn; UK Health Security Agency; The University of Manchester, Division of Population Health, Health Services Research & Primary Care, School of Health Sciences & Manchester Academic Health Science Centre<br>Cullen-Stephenson, Iona; University College London, Comprehensive Clinical Trials Unit<br>Fenner, Robert; University College London, Comprehensive Clinical Trials Unit<br>Verma, Arpana; The University of Manchester, Division of Population Health, Health Services Research & Primary Care, School of Health Sciences<br>Gordon, Adam; University of Nottingham, 12. Academic Unit of Injury, Recovery and Inflammation Sciences (IRIS), School of Medicine; NIHR, Applied Research Collaboration-East Midlands (ARC-EM)<br>Hopkins, Susan; UK Health Security Agency<br>Copas, Andrew; University College London, Institute for Global Health<br>Freemantle, Nick; University College London, Comprehensive Clinical

	Trials Unit<br>Flowers, Paul; University of Strathclyde, Department of Psychological Sciences and Health<br>Shallcross, Laura; University College London, Institute of Health informatics
**Primary Subject Heading**:	Infectious diseases
Secondary Subject Heading:	Epidemiology, Public health
Keywords:	COVID-19, Epidemiology < TROPICAL MEDICINE, Public health < INFECTIOUS DISEASES

SCHOLARONE™ Manuscripts

Title: Shaping care home COVID-19 testing policy: A Protocol for a pragmatic cluster randomised controlled trial of asymptomatic testing compared to standard care in care home staff (VIVALDI-CT).

Authors: Natalie Adams[1]; Oliver Stirrup[2]; James Blackstone[3]; Maria Krutikov[1]; Jackie Cassell[4,5]; Dorina Cadar[4,6]; Catherine Henderson[7]; Martin Knapp[7]; Lara Goscé[2,8]; Ruth Leiser[9]; Martyn Regan[5,10,11]; Iona Cullen-Stephenson[3]; Robert Fenner[3]; Aparna Verma[10]; Adam L Gordon[12,13]; Susan Hopkins[5]; Andrew Copas[2]; Nick Freemantle[3]; Paul Flowers[9] & Laura Shallcross[1].

1. Institute of Health informatics, UCL, London, UK
2. Institute for Global Health, UCL, London, UK
3. Comprehensive Clinical Trials Unit, Institute of Clinical Trials and Methodology, UCL, London, UK
4. Department of Primary Care and Public Health, Brighton and Sussex Medical School, Brighton, UK
5. UK Health Security Agency, London, UK
6. Centre for Dementia Studies, Department of Neuroscience, Brighton and Sussex Medical School, Brighton, UK
7. Care Policy and Evaluation Centre, London School of Economics and Political Science, London, UK
8. Department of Infectious Disease Epidemiology, London School of Hygiene and Tropical Medicine, London, UK
9. Department of Psychological Sciences and Health, University of Strathclyde, Glasgow, UK
10. Division of Population Health, Health Services Research & Primary Care, School of Health Sciences, The University of Manchester, Manchester, United Kingdom
11. Manchester Academic Health Science Centre, The University of Manchester, Manchester, United Kingdom.
12. Academic Unit of Injury, Recovery and Inflammation Sciences (IRIS), School of Medicine, University of Nottingham, Nottingham, UK
13. NIHR Applied Research Collaboration-East Midlands (ARC-EM), Nottingham, UK.

For peer review only - http://bmjopen.bmj.com/site/about/guidelines.xhtml

Corresponding author: Professor Laura Shallcross: l.shallcross@ucl.ac.uk

Word count: 4703/4000

Abstract

Introduction

Care home residents have experienced significant morbidity, mortality and disruption following outbreaks of SARS-CoV-2. Regular SARS-CoV-2 testing of care home staff was introduced to reduce transmission of infection, but it is unclear whether this remains beneficial. This trial aims to investigate whether use of regular asymptomatic staff testing, alongside funding to reimburse sick pay for those who test positive and meet costs of employing agency staff, is a feasible and effective strategy to reduce COVID-19 impact in care homes.

Methods and analysis

The VIVALDI-Clinical Trial (VIVALDI-CT) is a multi-centre, open label, cluster randomised controlled, phase III/IV superiority trial in up to 280 residential and/or nursing homes in England providing care to adults aged >65 years. All regular and agency staff will be enrolled, excepting those who opt out. Homes will be randomised to the intervention arm (twice weekly asymptomatic staff testing for SARS-CoV-2) or the control arm (current national testing guidance). Staff who test positive for SARS-CoV-2 will self-isolate and receive sick pay. Care providers will be reimbursed for costs associated with employing temporary staff to backfill for absence arising directly from the trial.

The trial will be delivered by a multi-disciplinary research team through a series of five work packages.

The primary outcome is incidence of COVID-19-related hospital admissions in residents. Secondary outcomes include the number and duration of outbreaks and home closures. Health economic and modelling analyses will investigate the cost-effectiveness and cost-

For peer review only - http://bmjopen.bmj.com/site/about/guidelines.xhtml

consequences of the testing intervention. A process evaluation using qualitative interviews will be conducted to understand intervention roll out and identify areas for optimisation to inform future intervention scale-up, should the testing approach prove effective and cost-effective. Stakeholder engagement will be undertaken to enable the sector to plan for results and their implications and to coproduce recommendations on the use of testing for policymakers.

Ethics and dissemination

The study has been approved by the London - Bromley Research Ethics Committee (reference number 22/LO/0846) and the Health Research Authority (HRA) (22/CAG/0165). The results of the trial will be disseminated regardless of the direction of effect. The publication of the results will comply with a trial-specific publication policy and will include submission to open access journals. A lay summary of the results will also be produced to disseminate the results to participants.

Trial registration details

The VIVALDI-CT was registered with the International Standard Randomised Controlled Trial Number website (ISRCTN 13296529) on 5 December 2022.

Strengths and limitations

- First trial to evaluate the benefits and harms of regularly testing care home staff for COVID-19 to protect residents from severe outcomes following infection
- Process evaluation, economic and modelling analyses will provide insights into intervention feasibility and costs/cost-effectiveness, informing future public health policy.
- The study demonstrates the potential for large scale trials in care homes that are delivered in partnership with care providers and capitalise on routinely collected data.
- The trial is being delivered in a rapidly changing policy and epidemiological context, which could undermine effective trial delivery.

For peer review only - http://bmjopen.bmj.com/site/about/guidelines.xhtml

Keywords

COVID-19; SARS-CoV-2; care homes; long-term care facility; epidemiology; asymptomatic testing; randomised controlled trial; public health; policy

For peer review only

For peer review only - http://bmjopen.bmj.com/site/about/guidelines.xhtml

Introduction

Context

In England, approximately 380,000 people (4% of > 65-year-olds) live in 11,000 care homes for older adults. Most care home residents ('residents') are older than 85 years, at least two-thirds live with dementia, and over half die within 12 months of admission to a care home.(1,2) Care home residents worldwide have experienced among the highest rates of COVID-19 mortality and morbidity,(3) and in England, they have also been subject to particularly strict and lengthy lock-down measures. Prolonged use of COVID-19 restrictions (e.g. social isolation, visitor restrictions) has had a devastating impact on residents' well-being, and their physical and mental health, for example depriving them of contact with family members in their final weeks of life.(4)

Current knowledge

Public health disease control measures were deployed rapidly and simultaneously in care homes early in the pandemic to reduce infection spread, limiting any assessment of the impact of individual measures. There have been no interventional studies of non-pharmaceutical control measures to reduce COVID-19 infection in care homes, but a Cochrane rapid review (published in September 2021) identified 11 observational and 11 modelling studies, all from high-income countries.(5) The review grouped interventions into entry regulations (e.g. reducing visitors), contact regulating and transmission-reducing measures (e.g. Personal Protective Equipment, PPE), surveillance (symptomatic and asymptomatic testing), and outbreak control measures. Across these domains the quality of evidence was poor. In addition, there was widespread recognition that some of these measures, such as preventing visitors from entering the care home, were associated with significant harm.

Throughout the pandemic, testing has been used in three ways to reduce transmission of infection: 1) symptomatic testing, 2) testing during outbreaks to reduce their duration and severity and 3) regular, asymptomatic testing.

For peer review only - http://bmjopen.bmj.com/site/about/guidelines.xhtml

In the UK, compliance with regular testing may have been driven by national policies incentivising testing, including with financial support (e.g., Adult Social Care Rapid Testing Fund introduced in January 2021, Infection Control Fund introduced May 2020).(6,7) However, relatively few published studies have examined how these influence compliance with asymptomatic testing in care homes. We conducted a rapid systematic review, spanning January 2020 to July 2022. It highlighted 14 international papers,(8–21) published in English. No studies used an experimental design, and none reported, or evaluated, interventions designed to improve compliance with SARS-CoV-2 testing. The papers used a range of designs (e.g., qualitative, cross-sectional quantitative, consensus building). Together these studies highlight the *multi-levelled* factors that have shaped adherence with SARS-CoV-2 testing in care homes. We then used the behaviour change wheel (22) as an approach to develop systematically potentially useful intervention content from the factors influencing testing identified within the literature. Subsequently, through a series of stakeholder engagement events with diverse care home staff and representatives from the care home sector, we agreed the content of a multi-level intervention designed to maintain compliance with twice weekly lateral flow device (LFD) testing for COVID-19 within intervention care homes ('Test to Care').

There remains a lack of evidence on whether the benefits of regular testing for COVID-19 outweigh its harms, and if so, under which scenarios. There have been no attempts in the literature to consolidate the considerable expertise and learning on how to ensure compliance with testing in this setting. From a policy perspective, the key question remains over appropriate thresholds for turning testing 'on' and 'off' in response to varying levels of 'COVID-19 threat' (e.g., high/low levels of infection in the community; the emergence of novel COVID-19 variants).

We posit that the best approach to address these questions is through a randomised clinical trial. Randomisation overcomes the problem of substantial heterogeneity among care homes as they vary in resident population, care provision, and uptake of control measures such as vaccination in staff, or use of facemasks, which limit the conclusions that can be drawn from observational studies. Although there are significant challenges associated with undertaking a trial in care homes in a changing policy and epidemiological context, there is

For peer review only - http://bmjopen.bmj.com/site/about/guidelines.xhtml

an urgent need for high-quality evidence to inform the future use of testing for SARS-CoV-2 and potentially other infections in this setting.

Study aims

We will investigate whether continued use of regular asymptomatic testing in staff is a feasible, effective, and cost-effective strategy to reduce the impact of COVID-19 in care homes. Findings will inform testing policy across the United Kingdom (UK) for COVID-19 and add to knowledge on the use of testing in care homes to prevent other respiratory viruses, such as influenza. These objectives will be delivered through a series of five interlinked work-packages (WPs) which are described in detail in Supplementary File 1.

Methods and analysis

Study Design

VIVALDI-CT is a multi-centre, open label, cluster randomised controlled, phase III/IV superiority trial.

Each eligible care home will be randomised to either standard care (SARS-CoV-2 testing policy for care home staff that is in place nationally at the time of trial operation), or regular asymptomatic testing of care home staff for COVID-19 using Lateral Flow Devices (LFDs) combined with support payments for sickness absence and agency staff backfill.

Study Setting

VIVALDI-CT will take place in up to 280 residential and/or nursing homes in England providing care to adults aged >65 years.

Recruitment

Due to rapid timescales for trial delivery, and the need to streamline and centralise data collection we will primarily partner with providers that manage multiple care homes. We will first contact the senior management teams of providers that we have previously worked with in the Vivaldi study (23) to determine if they are interested in trial participation.

For peer review only - http://bmjopen.bmj.com/site/about/guidelines.xhtml
For peer review only

Providers will be asked to supply a list of eligible care homes and confirm that the care home manager has provided consent for each listed home to participate. If we are unable to recruit sufficient homes from the Vivaldi network, we will work with provider representative organisations (e.g., National Care Forum, Care England, National Care Association) to identify other eligible providers.

Homes will be selected to capture diversity in care home size, population (nursing / residential / dementia care), ethnicity, geographical location, rural/urban and provider type (for-profit / not-for-profit). Inclusive participation will be a focus by ensuring larger and smaller care groups are included in trial, as well as focusing on diverse settings.

Inclusion and Exclusion Criteria

Only care home staff are eligible to participate in the testing intervention. This includes temporary (agency) staff with no restrictions i.e., catering, administrative, and maintenance staff, in addition to those in a resident-facing role. However, all care home staff, as well as residents, visitors, and relatives, are eligible to participate in interviews undertaken as part of the trial's process evaluation. All care home residents at participating homes are eligible for data collection and analysis of the outcomes specified.

Visitors, residents, and relatives are not eligible to take part in the testing intervention. Staff who visit the care home to provide care but are not employed by the care home e.g., GPs, health visitors, are not eligible to take part in either the interviews or the testing intervention.

Primary outcome

The primary outcome is the incidence of COVID-19 related hospital admissions in residents defined as admissions with a relevant International Statistical Classification of Diseases and Related Health Problems 10th revision (ICD-10) code (COVID hospitalisations to be defined as any hospital admission record with a primary or secondary ICD10 code of 'U071') and/or admissions in residents who test positive for COVID-19 within 24 hours following admission or in the 7 days before hospital admission. This is considered the most important outcome for policymakers.

For peer review only - http://bmjopen.bmj.com/site/about/guidelines.xhtml

Secondary outcomes

Although we have adopted a healthcare/NHS perspective for the primary outcome, we recognise the importance of capturing outcomes that are relevant to the social care sector, such as outbreaks and care home closures. This is reflected in our choice of secondary outcomes:

- Incidence rate of hospital admissions (all-cause) in residents for non-elective care, measured as events per 100,000 person-days of follow-up over the duration of the trial.
- Incidence rate of COVID-associated mortality in residents, measured as events per 100,000 person-days of follow-up over the duration of the trial.
- Incidence of all-cause mortality in residents, measured as events per 100,000 person-days of follow-up over the duration of the trial.
- Testing uptake in staff, measured as proportion of staff at each home participating in testing during each week of the trial.
- Prevalence of SARS-CoV-2 among staff who test, measured as proportion of staff with positive test result among those with at least one test recorded during each week of the trial.
- Incidence rate of SARS-CoV-2 infections detected in residents, measured as events per 100,000 person-days of follow-up over the duration of the trial.
- Incidence rate of home-level outbreaks, measured as events per 1000 days of follow-up over the trial duration.
- Duration of outbreaks, measured as days from first to last case within outbreaks occurring within the trial period.
- Incidence rate of care home closures due to outbreaks, measured as events per 1000 days of follow-up over the duration of the trial.
- Proportion of staff per home who are off sick during each week of the trial.
- Proportion of all shifts filled by agency staff at each home each week.
- Costs per test.
- Testing metrics e.g., staff time taken to conduct the test at work.
- The impact of testing on resident, staff and visitors e.g., Social Care related Quality of Life collected via interviews in WP3.

For peer review only - http://bmjopen.bmj.com/site/about/guidelines.xhtml

Sample Size

Based on observational data from the VIVALDI study we found that over the 3-month period of January-March 2022 1.8% of residents had a COVID-19 related hospital admission, and the Intra-Cluster Correlation (ICC) across homes was 0.003 (95% CI 0.000-0.007). We assume that we will observe a cumulative incidence of around 3.0% in the trial, which would require a trial duration of 5-6 months if the incidence rate is similar to that in winter 2021/2022, in combination with a conservative ICC value of up to 0.01 (higher in line with the higher cumulative incidence compared to 3 months), and an average care home size of 35 residents with coefficient of variation in size of 0.5. With a total of 280 homes randomised 1:1 to trial arms and taking the usual two-sided test at 5% significance level, the design provides 84% power to detect a reduction in COVID-19 related admissions due to intervention to 1.9% (relative risk 0.63).

Timeline

The trial programme will run from November 2022 to April 2024. The recruitment of care home providers and operation of the intervention will take place between December 2022 and March 2023, with the possibility that this will be extended if deemed necessary for data collection. Supplementary File 2 shows a participant timeline and Figure 1 is a schematic of the trial pathway.

Intervention allocation

Care homes will be randomised on a 1:1 ratio. If all providers are ready for trial participation at the same time, then all participating homes will be randomised at the same time. Otherwise, the homes from different providers will be randomised in a phased approach, as they become ready. Randomisation will be performed by the trial statistician based on pseudo-random number generation after trial enrolment and before intervention implementation. Restricted randomisation (specifically covariate constrained randomisation) will be used to ensure balance on care home provider, size, and region.

For peer review only - http://bmjopen.bmj.com/site/about/guidelines.xhtml

Blinding

Researchers and staff of participating care homes will not be blinded to their intervention allocation, as this would not be feasible.

Data collection

To facilitate trial set-up and minimise the burden on care home staff, much of the data for analysis will be obtained from routinely collected healthcare information held within the UK COVID-19 Datastore. (24) This will include results of LFD and PCR tests for SARS-CoV-2 for staff and residents, information on hospital admissions and deaths for residents, and vaccination history for residents. Data within the COVID-19 Datastore are linked to a pseudonymised ID at the level of each individual, which can be linked to Care Quality Commission-ID (CQC-ID), a unique ID number provided by the CQC to identify each care home, for participating care homes and associated staff or resident status. A new study specific pseudonymised ID will be created for each individual before export of data to UCL, in order to prevent any theoretical possibility of reidentification.

Hospital admissions data are linked to ICD-10 diagnostic codes (including COVID-19 codes) however, there is a lag of several months in the assignment of these codes. To allow timely monitoring of data quality for the primary outcome and limit the risk of omitting or double-counting hospital admissions in residents, during the intervention period providers will be asked to upload weekly lists of COVID-associated hospital admissions in residents from participating care homes to the COVID-19 Datastore. Linkage to individual pseudonymised IDs will allow comparison to the routinely collected hospital admission data once available.

To inform estimates of incidence rates which form the primary and secondary outcomes, we will collect the total number of residents in the home on a weekly basis from providers as this will allow us to estimate the denominator. We will also collect the total number of staff on a weekly basis from each home to inform estimates of testing uptake and explore the feasibility of collecting data on the number of staff who opt-out of asymptomatic testing (in the intervention arm).

Care home level (aggregate) data will be collected from providers on dates of care home closures, use of disease control measures, staff sickness absence and employment of agency

For peer review only - http://bmjopen.bmj.com/site/about/guidelines.xhtml

staff to inform health economic analyses. We will explore the feasibility of collecting care home level data on fees paid by residents who are funded by the local authority, and whether it is feasible to collect more detailed information on healthcare utilisation, such as primary care consultations and use of antivirals in residents.

Data on outbreak events (dates, size) will be obtained from the United Kingdom Health Security Agency (UKHSA) Adult Social Care Team. Data on the local incidence of COVID-19 and co-circulation of other respiratory viruses will be obtained from the UKHSA and/or the Office of National Statistics (ONS) Covid Infection Survey.

For peer review only

Data management

Individual-level trial data will be stored in the UCL Data Safe Haven (25) which is hosted by UCL. All identifiable data will be held only by individual care homes or NHS England (NHSE), who will act as Data Processor on behalf of UCL. These databases are protected by multi-layer firewalls with full data encryption at rest and in transit.

For qualitative interviews, data collection will occur remotely using secure communication methods and be conducted by University of Strathclyde (UoS) researchers. Upon completion of transcription at UoS, the pseudo-anonymised data will be stored on the UoS network in a secure, restricted access folder for 5 years from the time of end of trial. Raw data will be destroyed once transcription and quality checks have been performed. Consent forms obtained via interviews and focus groups in the process evaluation will also be stored securely.

Statistical analysis

Analysis of the primary outcome, and secondary outcomes expressed as event incidence, will be based on Poisson or negative binomial regression with cluster-robust standard errors, adjusting for calendar time and key care home characteristics used in the restricted randomisation such as provider, region, and size. We will also explore whether the intervention effect differs according to care home size, and other characteristics such as proportion of temporary staff. Unadjusted effect estimates from these analyses will be reported for completeness. Using interaction terms, we will explore whether the effect of the intervention on the primary outcome differed between time periods defined by the

For peer review only - http://bmjopen.bmj.com/site/about/guidelines.xhtml

national recommendations for testing in the routine care arm, should these change during data collection.

Analysis of the primary outcome will include all trial care homes (intention to treat analysis), and so represent a treatment policy estimand. We will also define an implementation score based on the frequency and proportion of staff testing at each home based on data the homes provide, which may vary over time. As an exploratory analysis we will assess whether the primary outcome is associated with this implementation score within the intervention arm and express the effect of the intervention relative to control arm for different levels of implementation. This analysis will be based on the same regression method as used for the primary analysis.

For peer review only

Health Economic Analysis

The health economic analysis will investigate the cost-effectiveness and cost-consequences of the testing intervention taking a NHS, Personal Social Services, and a societal perspective using a lifetime horizon (according to care home resident average age and life expectancy). The within-trial costs and outcomes in intervention and control groups will be examined from each perspective. Cost-effectiveness of the intervention in terms of the primary outcome and in terms of all-cause mortality will also be examined. Costs of admission will be excluded from the total costs under consideration in this case.

We will also examine cost-effectiveness in terms of the secondary outcomes of cases prevented and resident deaths prevented, and outcomes of hospital admission and number of outbreaks alongside costs offset/additional costs incurred in a cost-consequences analysis.

Process Evaluation

There is major diversity across care homes, for example in terms of provision of care, resident population, care home size, and the care home workforce. As a result, it is essential to consider the feasibility and sustainability of the intervention and how contextual factors might impact on the ability to scale it, if the trial suggests it is effective and cost-effective. These issues will be addressed in the process evaluation which aims to

For peer review only - http://bmjopen.bmj.com/site/about/guidelines.xhtml

understand intervention roll out and identify areas for optimisation to inform future intervention scale-up, should the testing approach prove effective and cost effective.

The objectives of the process evaluation are:

- To determine intervention acceptability.
- To determine the role of context in shaping the way, the intervention operated.
- To determine what can be learned about intervention fidelity and adaptation.
- To determine which intervention components worked as anticipated and which need further modification.
- To investigate unanticipated intervention effects.
- To determine what can be learned from the control group.

The process evaluation will develop implementation guidance and training packages ready for future scale up as well as details of minimal care home requirements and staff competencies necessary for intervention delivery.

Qualitative data will be collected from 28 (10%) care homes evenly distributed across each intervention and control arms and spaced across time.

Patient and Public Involvement

Patient public involvement (PPI) has already informed the development of this programme, by highlighting the barriers to testing and the need to capture its adverse impacts on staff, residents, and providers. Public advisors have also emphasised the importance of developing a strong plan for implementation, recognising the financial implications of long-term use of testing and sickness payments, informing our emphasis on implementation in WP5.

The PPI team will deliver the following objectives:

- To ensure that the 'voice and views' of the public regarding regular testing for COVID-19 are heard by the research team and the wider stakeholder group.
- To create an open, inclusive culture enabling effective communication between the study team, PPI group and the wider stakeholder and oversight groups.

For peer review only - http://bmjopen.bmj.com/site/about/guidelines.xhtml

- To agree an approach to communicate outputs from the trial to different audiences, including care home staff, residents and their families and the public using a variety of media.

Ethics and dissemination

Study monitoring

An independent Trial Steering Committee (TSC) and Data Monitoring and Ethics Committee (DMEC) will be formally responsible for programme oversight, ensuring the study is conducted in compliance with regulations. The DMEC will also be responsible for monitoring the accumulating data and making recommendations to the TSC on whether the trial should continue as planned.

A Trial Management Group (TMG) will be responsible for the design, coordination, and strategic management of the trial.

Safety reporting

Staff at the sites randomised to asymptomatic testing will report the occurrence of Serious Adverse Events (SAEs) considered ‘related’ to the intervention only. In such cases site personnel will complete a Serious Adverse Event (SAE) report within 24 hours of notification of the event. Clinical review of any SAEs will take place and be reported to the Research Ethics Committee if deemed both ‘related’ to the trial intervention and ‘unexpected’ in line with UK Health Research Authority non-drug trial reporting requirements.

Research ethics approval

The study has been approved by the London - Bromley Research Ethics Committee (reference number 22/LO/0846) and the Health Research Authority (HRA) (22/CAG/0165).

Consent and opt out

Care home providers and home managers will be asked if their care home(s) are willing to participate in the trial. High staff turnover, in conjunction with the large number of care

For peer review only - http://bmjopen.bmj.com/site/about/guidelines.xhtml
For peer review only

homes participating in the trial, means that it is not feasible to obtain individual consent from staff or regarding the use of testing data. Staff and residents have the option of opting out from the processing and analysis of their individual-level data within this study at any time during the study.

Identifiable data submitted by care homes as part of the study will be pseudonymised by NHS England before it is provided to the research team. This study has section 251 support to allow the disclosure of confidential patient information (regarding testing in staff) from care homes to NHS England, for the purposes of monitoring uptake of the testing in the control and intervention arms of the trial.

The study will also collect limited individual-level identifiable data from residents to ensure the primary outcome can be determined accurately. It is not feasible to seek individual-level consent from every resident for the use of these data due to high levels of cognitive impairment in residents and excluding data from a large proportion of residents would compromise the scientific value of the trial and the subsequent generalisability of trial findings. This study has section 251 support to allow the disclosure of confidential patient information (regarding residents admitted to hospital) from care homes to NHS England, for the purposes of linkage to the COVID-19 datastore and to enable NHS England to use confidential patient information from SARS-CoV-2 tests to link to other NHS datasets within the COVID-19 datastore.

Care home managers in the subset of homes selected for qualitative data collection (focus groups or one to one interviews) will be asked to disseminate recruitment materials to staff within the home via word of mouth, email, or other routine modes of communication. Upon receipt of staff contact details, interested staff will then be sent participant information sheets about the study, given the option to ask questions about the study, complete on-line consent forms and provide brief sociodemographic details to enable the study team to monitor total sample composition. Having checked on-line consent has already been given and after exploring any remaining unanswered questions raised by the PIS, the participants will be asked to also give recorded oral consent to participate.

For peer review only

For peer review only - http://bmjopen.bmj.com/site/about/guidelines.xhtml

Confidentiality

All data will be handled in accordance with the Data Protection Act 2018, the UK General Data Protection Regulation (UK GDPR) and subsequent updates and amendments.

Dissemination policy

The results of the trial will be disseminated regardless of the direction of effect. The publication of the results will comply with a trial-specific publication policy and will include submission to open access journals.

A lay summary of the results will also be produced to disseminate the results to participants. A summary of results will be included online in the publicly accessible Health Research Authority (HRA) website within 12 months of date of trial closure. A Statistical Analysis Plan will also be published under open access arrangements.

Discussion

The proposed research faces several specific methodological and operational challenges, primarily because the study is being conducted at pace in a dynamic epidemiological and policy context. We outline the main challenges and potential mitigations here.

In designing this trial, we worked closely with care home staff and providers to design a testing intervention that would be feasible and acceptable. In particular, we worked closely with each care provider participating in the study to understand how they process and organise sick pay and employ agency staff, and the likely costs. We met with the providers' HR teams and also members of their senior management team. We established a flexible approach to reimbursing sick pay and agency staff to ensure all legitimate costs will be covered. Providers will be asked to provide evidence of the actual costs accrued each month and this will be verified by the funder before payments are released, ensuring that compensation for staff sickness is adequate.

We have also organised a series of stakeholder engagement events with diverse care home staff and representatives from the care home sector to agree the content of the intervention, which has been designed to maintain compliance with testing and ensure staff in intervention care homes will not be disadvantaged. Engagement events have included

For peer review only - http://bmjopen.bmj.com/site/about/guidelines.xhtml
For peer review only

consideration of testing acceptability, how to increase uptake of testing and logistics related to support payments, such as when and how they should be paid to staff. These events will also provide an opportunity to discuss concerns related to potential consequences for staff and organisations as a result of participating in the trial. All these issues were taken into account when designing the testing protocol and associated support payments. Care home staff in participating care homes were noted to be extremely familiar with the process of regular asymptomatic testing and reimbursement of sick pay as this was in place throughout the pandemic in England.

There is a significant question over whether the trial will be sufficiently powered to detect a statistically significant outcome. This is predicated on the both the epidemiological event rate during the intervention period, and willingness of sufficient numbers of care homes to participate.

We are acutely aware that there is a risk that rates of COVID-19 might decline from estimates used from previous years (making it impossible to achieve significance for the primary outcome), but we have taken the view that this is unique opportunity to try to generate data on the effectiveness, benefits and harms of regularly testing asymptomatic staff for COVID-19 (with financial support for staff sickness and agency staff backfill) to prevent severe outcomes in residents, which would be lost if we did not attempt this trial. At the time of trial design and the application for funding (August 2022) it was very unclear whether there would be a resurgence of COVID-19 in autumn/winter associated with the emergence of a new variant.

In the event that the trial's primary outcome cannot be delivered as planned, the non-trial work packages will still generate valuable evidence to inform future use of testing in care homes, by characterising barriers and facilitators to testing, estimating costs of the testing intervention and generating models that can be used to estimate the impact of testing on infections and hospital admissions under different epidemiological scenarios.

Whilst we considered including testing for visitors as part of the intervention, this would have introduced further complexity regarding consent. It would also have strayed from the approach that was adopted during the pandemic in England, which is what we wanted to evaluate.

For peer review only - http://bmjopen.bmj.com/site/about/guidelines.xhtml

We note that results of a trial investigating the benefits of COVID-19 related staff testing and sickness support payments in a care home context would have been beneficial earlier in the course of the pandemic. The challenge in trying to do this has been that it would have been extremely difficult to persuade public health agencies (in the UK or elsewhere) that it was reasonable to withhold regular asymptomatic testing in control homes when there were high levels of COVID-19 transmission in the community. We feel it remains a highly salient research question and the results should help inform policy for care home preparedness, both in relation to COVID-19 but also in support of future research into wider institutional infectious disease transmission mitigation.

Whilst there are significant methodological challenges to conducting this study, it is our view that we need to learn how to do trials at pace and scale in care homes, to improve the quality of care for residents. In addition to generating important evidence on the effectiveness, benefits and harms of asymptomatic testing for COVID-19 in staff to prevent severe outcomes in residents, this trial will provide important learning to inform the design and delivery of future care home trials.

Trial Registration and Reporting Guidelines

The VIVALDI-CT was registered with the International Standard Randomised Controlled Trial Number website (ISRCTN 13296529) (26) on 5 December 2022 and the protocol adheres to the SPIRIT 2013 Statement (27).

Author Statement

NA, JB, MKr, LS and PF developed the study protocol and contributed to the writing of the manuscript. OS and AC were involved in development of the statistical analysis facets of the trial and contributed to the writing of the manuscript. CH and MKn were involved in development of the health economic analysis facets of the study and contributed to the writing of the manuscript. RL was involved in development of the process evaluation of the study and contributed to the writing of the manuscript. DC and JC were involved in development of the health-related quality of life facets of the study and contributed to the writing of the manuscript. LG was involved in development of the economic modelling of the study and contributed to the writing of the manuscript. ICS is the trial manager for the

For peer review only - http://bmjopen.bmj.com/site/about/guidelines.xhtml

study. RF will oversee data collection and management. MR, AV, AG, NF and SH are co-applicants responsible for supporting the operationalisation of the study. LS and PF are the co- chief investigators for the study. All authors critically reviewed and approved the final version.

Funding

This work is supported by the NIHR Health and Social Care Delivery Research (HSDR) Programme number [154310]. Costs associated with SARS-CoV-2 testing including support payments for care home staff and for care homes to fund agency staff backfill will be funded by the UK Health Security Agency (UKHSA).

Sponsor

The VIVALDI-CT is sponsored by University College London, represented by the UCL Comprehensive Clinical Trials Unit (UCL CCTU).

Competing interests

None declared.

Study Status

Current Protocol – Version 3.0 dated 04 Jan 2023. At the time of protocol submission, recruitment of sites had been completed.

Data Statement

No data are associated with this article.

Data from the trial will be available for sharing between the trial researchers as detailed in the Data Sharing Agreement between the institutions hosting the VIVALDI-CT researchers.

Requests for access to trial data will be considered, and approved in writing where appropriate, after formal application to the TSC. Considerations for approving access are documented in the TSC Terms of Reference.

For peer review only

For peer review only - http://bmjopen.bmj.com/site/about/guidelines.xhtml

Acknowledgements

We are grateful to the care homes who have already agreed to participate in the VIVALDI-CT. We would also like to acknowledge the role of the care homes participating in the VIVALDI Study for sharing their learning from the pandemic with us.

We also acknowledge the support of the independent members of the Trial Steering Committee (TSC):

- Prof Alistair Hay (University of Bristol)
- Dr Jennifer Thompson (LSHTM)
- Dr Michael Larkin (Aston University)
- Zoe Fry (Outstanding Society)
- Samantha Crawley (Bracebridge)
- Margaret Ogden (independent PPI member)

As well as the Data Monitoring and Ethics Committee (DMEC):

- Prof Karla Hemming (University of Birmingham)
- Dr Tania Kalsi (Guy's and St Thomas' NHS Foundation Trust)
- Dr Terry Quinn (University of Glasgow)

We would also like to thank members of the UKHSA:

- Dr Tom Fowler (UKHSA)
- Alex Barton (DHSC)
- Prof Jackie Cassell (UKHSA)
- Dr Sarah Tunkel (UKHSA)

For peer review only - http://bmjopen.bmj.com/site/about/guidelines.xhtml

Figures and tables

Supplementary file 1: Description of work packages in the VIVALDI-CI

Supplementary file 2: Participant timeline

For peer review only

For peer review only - http://bmjopen.bmj.com/site/about/guidelines.xhtml

Figure Legend

Figure 1: Flow diagram of trial pathway

For peer review only

For peer review only - http://bmjopen.bmj.com/site/about/guidelines.xhtml

References

1. Kinley J, Hockley J, Stone L, et al. The provision of care for residents dying in UK nursing care homes. *Age Ageing*. 2014;43(3):375-379. doi:10.1093/ageing/aft158

2. Alzheimer's Society. *Dementia UK Update*.; 2014. Accessed November 27, 2021. https://www.alzheimers.org.uk/sites/default/files/migrate/downloads/dementia_uk_update.pdf

3. Levin AT, Jylhävä J, Religa D, Shallcross L. COVID-19 prevalence and mortality in longer-term care facilities. *Eur J Epidemiol*. 2022;37(3):227-234. doi:10.1007/s10654-022-00861-w

4. Kaelen S, van den Boogaard W, Pellecchia U, et al. How to bring residents' psychosocial well-being to the heart of the fight against Covid-19 in Belgian nursing homes—A qualitative study. Kotozaki Y, ed. *PLOS ONE*. 2021;16(3):e0249098. doi:10.1371/journal.pone.0249098

5. Stratil JM, Biallas RL, Burns J, et al. Non-pharmacological measures implemented in the setting of long-term care facilities to prevent SARS-CoV-2 infections and their consequences: a rapid review. Cochrane Public Health Group, ed. *Cochrane Database Syst Rev*. 2021;2021(9). doi:10.1002/14651858.CD015085.pub2

6. Adult Social Care Rapid Testing Fund. GOV.UK. Accessed October 7, 2022. https://www.gov.uk/government/publications/adult-social-care-rapid-testing-fund

7. Adult Social Care Infection Control Fund. GOV.UK. Accessed July 26, 2022. https://www.gov.uk/government/publications/adult-social-care-infection-control-fund

8. Gray KL, Birtles H, Reichelt K, James IA. The experiences of care home staff during the COVID-19 pandemic: A systematic review. *Aging Ment Health*. 2022;26(10):2080-2089. doi:10.1080/13607863.2021.2013433

9. Pétré B, Paridans M, Gillain N, et al. Factors influencing the adoption and participation rate of nursing homes staff in a saliva testing screening programme for COVID-19. *PloS One*. Published online June 30, 2022. doi:10.1371/journal.pone.0270551

10. Pétré B, Paridans M, Gillain N, et al. Acceptability of Community Saliva Testing in Controlling the COVID-19 Pandemic: Lessons Learned from Two Case Studies in Nursing Homes and Schools. *Patient Prefer Adherence*. 2022;16:625-631. doi:10.2147/PPA.S349742

11. Walker, J. Implementing COVID-19 infection prevention and control measures in long-term care settings. *Nurs Older People*. Published online April 5, 2022. doi:10.7748/nop.2022.e1384

12. Nyashanu M, Pfende F, Ms E. Triggers of mental health problems among frontline healthcare workers during the COVID-19 pandemic in private care homes and

For peer review only - http://bmjopen.bmj.com/site/about/guidelines.xhtml

domiciliary care agencies: Lived experiences of care workers in the Midlands region, UK. *Health Soc Care Community*. Published online February 2022. doi:10.1111/hsc.13204

13. Tulloch J, Micocci M, Buckle P, et al. Enhanced lateral flow testing strategies in care homes are associated with poor adherence and were insufficient to prevent COVID-19 outbreaks: results from a mixed methods implementation study. *Age Ageing*. Published online November 10, 2021. doi:10.1093/ageing/afab162

14. Kierkegaard P, Micocci M, McLister A, et al. Implementing lateral flow devices in long-term care facilities: experiences from the Liverpool COVID-19 community testing pilot in care homes- a qualitative study. *BMC Health Serv Res*. Published online October 25, 2021. doi:10.1186/s12913-021-07191-9

15. Oldfield B, DeCosta S, Petterson L, Lagarde S, Olson D. A Blueprint for Community Health Center and Nursing Home Partnership: Testing for COVID-19 among Residents and Staff at Long-term Care Facilities. *J Health Care Poor Underserved*. Published online 2021. doi:10.1353/hpu.2021.0001

16. Micocci M, Gordon A, Allen A, et al. COVID-19 testing in English care homes and implications for staff and residents. *Age Ageing*. Published online May 5, 2021. doi:10.1093/ageing/afab015

17. White E, Wetle T, Reddy A, Baier R. Front-line Nursing Home Staff Experiences During the COVID-19 Pandemic. *J Am Med Dir Assoc*. Published online January 2021. doi:10.1016/j.jamda.2020.11.022

18. Ouslander J, Grabowski D. COVID-19 in Nursing Homes: Calming the Perfect Storm. *J Am Geriatr Soc*. Published online October 2020. doi:10.1111/jgs.16784

19. Wasserman M, Ouslander J, Lam A, et al. Editorial: Diagnostic Testing for SARS-Coronavirus-2 in the Nursing Facility: Recommendations of a Delphi Panel of Long-Term Care Clinicians. *J Nutr Health Aging*. Published online 2020. doi:10.1007/s12603-020-1401-9

20. Gordon A, Goodman C, Achterberg W, et al. Commentary: COVID in care homes-challenges and dilemmas in healthcare delivery. *Age Ageing*. Published online August 24, 2020. doi:10.1093/ageing/afaa113

21. COVID-19 and policies for care homes in the first wave of the pandemic in European welfare states: Too little, too late? - Mary Daly, Margarita León, Birgit Pfau-Effinger, Costanzo Ranci, Tine Rostgaard, 2022. Accessed July 26, 2022. https://journals.sagepub.com/doi/full/10.1177/09589287211055672

22. Michie S, van Stralen MM, West R. The behaviour change wheel: A new method for characterising and designing behaviour change interventions. *Implement Sci*. 2011;6(1):42. doi:10.1186/1748-5908-6-42

For peer review only - http://bmjopen.bmj.com/site/about/guidelines.xhtml

23. Krutikov M, Palmer T, Donaldson A, et al. Study Protocol: Understanding SARS-Cov-2 infection, immunity and its duration in care home residents and staff in England (VIVALDI). *Wellcome Open Res*. 2021;5:232. doi:10.12688/wellcomeopenres.16193.2

24. NHS England. NHS COVID-19 Data Store. Accessed October, 7 2022. https://www.england.nhs.uk/contact-us/privacy-notice/how-we-use-your-information/covid-19-response/nhs-covid-19-data-store/

25. UCL. Data Safe Haven (DSH). Information Services Division. Published February 14, 2018. Accessed October 7, 2022. https://www.ucl.ac.uk/isd/services/file-storage-sharing/data-safe-haven-dsh

26. Shallcross L, Shallcross L, Enquiries C. Regular asymptomatic COVID-19 testing in care home staff. doi:10.1186/ISRCTN13296529

27. Chan AW, Tetzlaff JM, Altman DG, et al. SPIRIT 2013 statement: defining standard protocol items for clinical trials. *Ann Intern Med*. 2013;158(3):200-207. doi:10.7326/0003-4819-158-3-201302050-00583

For peer review only

For peer review only - http://bmjopen.bmj.com/site/about/guidelines.xhtml

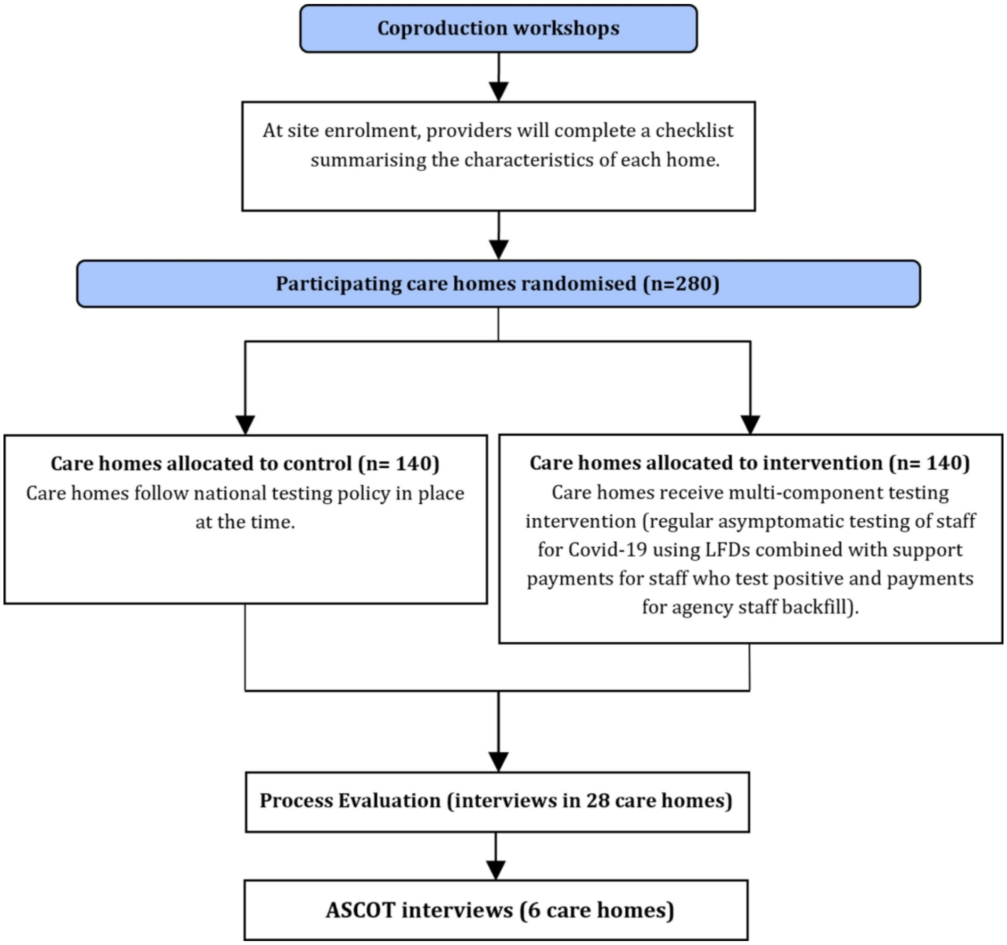

Figure 1: Flow diagram of trial pathway

166x156mm (330 x 330 DPI)

Supplementary File 1: Description of work packages in the VIVALDI-CT

Work Package	Aims	Participants	Methods	Data collection	Data analysis	Outcomes
WP1	To coproduce a sustainable testing intervention for care home staff.	Care home staff, providers and policymakers.	A series of workshops with care home stakeholders (e.g., home managers, staff, providers) and policymakers (including those with knowledge of testing logistics).<br><br>A workshop will also take place with residents with capacity to consent, relatives and visitors.	1) Consolidation of existing insights into routine testing gleaned through past experiences with the COVID-19 pandemic; 2) discussion of the intervention prototype; and 3) Operationalisation of intervention in ways which are likely to be acceptable and appropriate within the sector.	Data (transcript, detailed notes, images) will be analysed thematically to further develop the initial programme theory specifying important elements of the context, key intervention components, their mechanisms, and their relation to a hierarchy of outcome measures (e.g., health, social and financial outcomes).	Development of a testing intervention for use in WP2
WP2	To evaluate the effectiveness of the testing intervention compared to the recommended testing protocol that is in place at the time in a pragmatic cluster randomised trial.	Care home staff.	A cluster randomised controlled trial in which asymptomatic care home staff will be randomised at provider level to either test twice weekly using LFDs or to follow current national testing guidance.	At enrolment, providers will complete a checklist summarising characteristics of each participating care home. Each week during the testing period care home managers will be asked to record: 1) dates of hospital admissions for residents and their corresponding NHS	Analysis of the primary outcome, and secondary outcomes expressed as event incidence, will be based on Poisson or negative binomial regression with cluster-robust standard errors, adjusting for calendar time and key care home characteristics used in the restricted	The primary outcome will be the incidence of COVID-19 related hospital residents.

				numbers and/or identifiers, 2) number of staff and residents in each care home. The study will collect limited individual-level identifiable data from residents to ensure that the primary outcome of hospital admissions is accurate. Linkage to other routine datasets (hospitalisations, cause of death, COVID-19 test results, vaccination status).	randomisation such as provider, region, and size.	
WP3A	To understand the intervention roll out and identify areas for optimisation to inform future intervention scale-up, should the testing approach prove effective and cost effective	Care home staff	The intervention programme theory and associated logic models from WP1 will be used to undertake a parallel mixed methods process evaluation.<br>Interviews and focus groups will be undertaken with staff.	Data collection will be facilitated though virtual platforms (according to participant norms).<br><br>Quantitative data will include descriptive statistics from across all trial sites complemented by multivariate analyses of data sets where appropriate.	A combination of deductive and inductive thematic analysis will be used.<br><br>Interview/focus group transcriptions and qualitative survey data will be imported into NVivo10 software to facilitate data handling, organisation and coding. Analysis will firstly be	Implementation guidance and training packages ready for future scale up;<br><br>Details of minimal care home requirements and staff competencies necessary for

For peer review only

				Qualitative data will be collected from 28 (10%) care homes evenly distributed across each intervention and control arms and spaced across time. Selection criteria will focus on geography, socio-economic status of area, highest/lowest rates of infection. Heterogeneous samples of staff will be recruited. After consent is attained qualitative data will be collected primarily using focus groups. One to one interviews will also be possible if requested.	thematic and use a combination of deductive and inductive thematic analysis.	intervention delivery.
WP3B	To conduct a mixed methods, process evaluation and exploratory analysis to explore the impact of testing and outbreaks on social care related quality of life in home care residents	Care home residents	The Adult Social Care Outcomes Toolkit (ASCOT) will be used to assess the impact of the intervention and outbreaks on residents. ASCOT is a well-established tool for measuring social care related quality of life	In person interviews. If in-person resident interviews are not possible, Essential Caregivers (designated visitor allowed even during outbreaks) or members of staff will be interviewed.	The ASCOT tool will be used to assess SCRQoL at baseline, during/immediately after an outbreak or mid study, and at end of the study. ASCOT data will be combined with individual level demographic and health and aggregate	Outputs will also be integrated with findings from WP3A to provide a holistic assessment of the acceptability and feasibility of the testing intervention.

For peer review only

			(SCRQoL). We will establish an ASCOT cohort of six care homes stratified by type and size, distributed equally across control and intervention arms. Homes taking part in the qualitative interviews in WP3A are excluded.		home level data to give a comprehensive description of SCRQoL.	
WP4A	To evaluate the costs and benefits of the testing intervention.	NHS, providers, residents, families and staff.	We will examine within-trial costs and outcomes in intervention and control groups from each perspective. We will examine the cost-effectiveness of the intervention in terms of the primary outcome and in terms of all-cause mortality. The costs of admission will be excluded from the total costs under consideration in this case. We will also examine cost-effectiveness in terms of the secondary outcomes	We will examine the cost-effectiveness of the intervention in terms of the primary outcome, excluding cost of admission from total cost. We will also examine cost-effectiveness in terms of secondary outcomes of cases prevented and resident deaths prevented. We will examine outcomes of hospital admission and number of outbreaks alongside costs offset/additional costs	Generalised linear models appropriate to counts (hospital admissions, numbers of outbreaks, cases) or binary outcomes (deaths) and costs will be applied. For the purposes of the cost-effectiveness analyses, these will take into account possible correlations between costs and outcomes either by non-parametric bootstrapping of separate regressions or joint modelling approaches such as seemingly unrelated	Cost-effectiveness of the intervention in terms of the primary trial outcome (incidence of COVID-19 related hospital admission in residents.)<br><br>Cost-effectiveness in terms of the secondary outcomes of cases prevented and resident

For peer review only - http://bmjopen.bmj.com/site/about/guidelines.xhtml

			of cases prevented and resident deaths prevented. We will examine the outcomes of hospital admission and number of outbreaks alongside costs offset/additional costs incurred in a cost-consequences analysis.	incurred in a cost-consequences analysis. investigate the cost-effectiveness and cost-consequences of the testing intervention taking both a NHS, Personal Social Services, and a societal perspective.	regressions. Where individual level data are available, analyses will take a multilevel approach to adjust for clustering at the care home level either by two-stage bootstrapping of separate regressions or simultaneous modelling. Incremental cost-effectiveness ratios will be presented, net benefit calculated over a range of willingness to pay values for gains in outcomes to generate cost-effectiveness acceptability curves.	deaths prevented.<br><br>Cost-consequences of hospital admission and number of outbreaks alongside costs offset/additional costs incurred.
WP4B	To model costs and benefits of the testing intervention under different scenarios	NHS, providers, residents, families and staff.	A compartmental model will be built to study transmission of infection and infer the proportion of COVID-19 infections and deaths averted by the intervention under different epidemiological	Data from WP4A will be used.	The model will consider two populations i.e., home residents and staff, and take into account both symptomatic and asymptomatic cases, as well as hospital admissions and deaths.	Estimate of the projected cost-effectiveness of the intervention.

For peer review only

			scenarios (e.g., high/low community incidence of infection, care home population size etc).<br><br>Two scenarios will be modelled: 1) standard care: residents and staff get tested only if they show symptoms; and 2) intervention: standard care plus regular testing of all staff for COVID-19.		The model will be calibrated to trial results and modelling results will be projected in time by extending the time horizon. Unit costs calculated in the first part of the economic analysis will be discounted to future years values and associated to modelling results to estimate the projected cost-effectiveness of the intervention.<br><br>We will also explore the short and long-term costs of the testing intervention under different epidemiological scenarios (e.g., high/low community incidence of infection).	
WP5	To coproduce recommendations on the use of	Providers, care home staff, the NHS and	Three round-table discussions for a	Discussions will be based on world café methodological	Data (transcript, detailed notes, images) will be analysed thematically.	Formal mechanism to ensure

For peer review only

	regular testing for policymakers	primary care, policymakers and public health teams at national and regional / local level, community, residents and families.	maximum of 20 stakeholders.	principles. The views of the community, residents and families will be represented through organisations such as Healthwatch, the Residents and Relatives association and the VIVALDI-CT PPI group.		stakeholders are aware and involved in the work as it progresses and to enable the sector to prepare and plan for results and their implications.<br><br>Production of recommendations on the use of regular testing for policymakers.

For peer review only

Supplementary File 2: Participant Timeline

Trial visit number	Baseline	Care home data collection period							
**Month**	**Month 0**	**Months 1-4**		**Survey**	**Routine COVID-19 datastore**	**Uploaded to Foundry by provider data manager**	**To be reported as and when occurs**	**Weekly collection from providers**	**Participants**
		**Sites on Intervention arm**	**Sites on Standard Care arm**						
Demography	x			x	x				
Resident registry (patient type)	x			x					
Vaccination status	x				x				
Care home characteristics	x			x	x				
Number of residents per home (weekly)		x	x					x	
COVID-associated hospital admission events in residents		x	x		x	x			
Number of staff absent from work		x	x					x	
Total shifts at home this week (sum for all staff)		x	x					x	
Number of agency shifts		X	x					x	

Number of staff per home (weekly)		x	x					x		
Number of staff opting out of asymptomatic testing (weekly)*		x						x		
Routine data on LFD and PCR tests (staff+residents)		x	x		x					
Routine data on hospital admissions (residents)		x	x		x					
Routine data on mortality (residents)		x	x		x					
Outbreak event data		x	x					x		
Safety reporting		x	x				x	x		
Process Evaluation (3a)										
Focus groups		x	x						x	
Interviews		x	x						x	

* If the control arm starts routine testing, then collected from both arms

For peer review only

STANDARD PROTOCOL ITEMS: RECOMMENDATIONS FOR INTERVENTIONAL TRIALS

SPIRIT 2013 Checklist: Recommended items to address in a clinical trial protocol and related documents*

Section/item	Item No	Description	Page No
**Administrative information**			
Title	1	Descriptive title identifying the study design, population, interventions, and, if applicable, trial acronym	1
Trial registration	2a	Trial identifier and registry name. If not yet registered, name of intended registry	16
	2b	All items from the World Health Organization Trial Registration Data Set	1-17
Protocol version	3	Date and version identifier	17
Funding	4	Sources and types of financial, material, and other support	16
Roles and responsibilitie s	5a	Names, affiliations, and roles of protocol contributors	16-18
	5b	Name and contact information for the trial sponsor	16
	5c	Role of study sponsor and funders, if any, in study design; collection, management, analysis, and interpretation of data; writing of the report; and the decision to submit the report for publication, including whether they will have ultimate authority over any of these activities	16,17
	5d	Composition, roles, and responsibilities of the coordinating centre, steering committee, endpoint adjudication committee, data management team, and other individuals or groups overseeing the trial, if applicable (see Item 21a for data monitoring committee)	16-18
**Introduction**			
Background and rationale	6a	Description of research question and justification for undertaking the trial, including summary of relevant studies (published and unpublished) examining benefits and harms for each intervention	4-5
	6b	Explanation for choice of comparators	4-5
Objectives	7	Specific objectives or hypotheses	5-6

For peer review only - http://bmjopen.bmj.com/site/about/guidelines.xhtml

Trial design	8	Description of trial design including type of trial (eg, parallel group, crossover, factorial, single group), allocation ratio, and framework (eg, superiority, equivalence, noninferiority, exploratory)	6

Methods: Participants, interventions, and outcomes

Study setting	9	Description of study settings (eg, community clinic, academic hospital) and list of countries where data will be collected. Reference to where list of study sites can be obtained	6
Eligibility criteria	10	Inclusion and exclusion criteria for participants. If applicable, eligibility criteria for study centres and individuals who will perform the interventions (eg, surgeons, psychotherapists)	7
Interventions	11a	Interventions for each group with sufficient detail to allow replication, including how and when they will be administered	9
	11b	Criteria for discontinuing or modifying allocated interventions for a given trial participant (eg, drug dose change in response to harms, participant request, or improving/worsening disease)	13
	11c	Strategies to improve adherence to intervention protocols, and any procedures for monitoring adherence (eg, drug tablet return, laboratory tests)	6
	11d	Relevant concomitant care and interventions that are permitted or prohibited during the trial	6
Outcomes	12	Primary, secondary, and other outcomes, including the specific measurement variable (eg, systolic blood pressure), analysis metric (eg, change from baseline, final value, time to event), method of aggregation (eg, median, proportion), and time point for each outcome. Explanation of the clinical relevance of chosen efficacy and harm outcomes is strongly recommended	7-8
Participant timeline	13	Time schedule of enrolment, interventions (including any run-ins and washouts), assessments, and visits for participants. A schematic diagram is highly recommended (see Figure)	9, Table 2
Sample size	14	Estimated number of participants needed to achieve study objectives and how it was determined, including clinical and statistical assumptions supporting any sample size calculations	9
Recruitment	15	Strategies for achieving adequate participant enrolment to reach target sample size	6

Methods: Assignment of interventions (for controlled trials)

Allocation:

For peer review only - http://bmjopen.bmj.com/site/about/guidelines.xhtml

Sequence generation	16a	Method of generating the allocation sequence (eg, computer-generated random numbers), and list of any factors for stratification. To reduce predictability of a random sequence, details of any planned restriction (eg, blocking) should be provided in a separate document that is unavailable to those who enrol participants or assign interventions	9
Allocation concealment mechanism	16b	Mechanism of implementing the allocation sequence (eg, central telephone; sequentially numbered, opaque, sealed envelopes), describing any steps to conceal the sequence until interventions are assigned	9
Implementation	16c	Who will generate the allocation sequence, who will enrol participants, and who will assign participants to interventions	9
Blinding (masking)	17a	Who will be blinded after assignment to interventions (eg, trial participants, care providers, outcome assessors, data analysts), and how	10
	17b	If blinded, circumstances under which unblinding is permissible, and procedure for revealing a participant's allocated intervention during the trial	N/A
**Methods: Data collection, management, and analysis**			
Data collection methods	18a	Plans for assessment and collection of outcome, baseline, and other trial data, including any related processes to promote data quality (eg, duplicate measurements, training of assessors) and a description of study instruments (eg, questionnaires, laboratory tests) along with their reliability and validity, if known. Reference to where data collection forms can be found, if not in the protocol	10-11
	18b	Plans to promote participant retention and complete follow-up, including list of any outcome data to be collected for participants who discontinue or deviate from intervention protocols	10-11
Data management	19	Plans for data entry, coding, security, and storage, including any related processes to promote data quality (eg, double data entry; range checks for data values). Reference to where details of data management procedures can be found, if not in the protocol	11
Statistical methods	20a	Statistical methods for analysing primary and secondary outcomes. Reference to where other details of the statistical analysis plan can be found, if not in the protocol	11-12
	20b	Methods for any additional analyses (eg, subgroup and adjusted analyses)	11-12
	20c	Definition of analysis population relating to protocol non-adherence (eg, as randomised analysis), and any statistical methods to handle missing data (eg, multiple imputation)	10-11

Methods: Monitoring

Data monitoring	21a	Composition of data monitoring committee (DMC); summary of its role and reporting structure; statement of whether it is independent from the sponsor and competing interests; and reference to where further details about its charter can be found, if not in the protocol. Alternatively, an explanation of why a DMC is not needed	13
	21b	Description of any interim analyses and stopping guidelines, including who will have access to these interim results and make the final decision to terminate the trial	13
Harms	22	Plans for collecting, assessing, reporting, and managing solicited and spontaneously reported adverse events and other unintended effects of trial interventions or trial conduct	13
Auditing	23	Frequency and procedures for auditing trial conduct, if any, and whether the process will be independent from investigators and the sponsor	13

Ethics and dissemination

Research ethics approval	24	Plans for seeking research ethics committee/institutional review board (REC/IRB) approval	14
Protocol amendments	25	Plans for communicating important protocol modifications (eg, changes to eligibility criteria, outcomes, analyses) to relevant parties (eg, investigators, REC/IRBs, trial participants, trial registries, journals, regulators)	16
Consent or assent	26a	Who will obtain informed consent or assent from potential trial participants or authorised surrogates, and how (see Item 32)	14-15
	26b	Additional consent provisions for collection and use of participant data and biological specimens in ancillary studies, if applicable	14-15
Confidentialit y	27	How personal information about potential and enrolled participants will be collected, shared, and maintained in order to protect confidentiality before, during, and after the trial	15
Declaration of interests	28	Financial and other competing interests for principal investigators for the overall trial and each study site	17
Access to data	29	Statement of who will have access to the final trial dataset, and disclosure of contractual agreements that limit such access for investigators	17
Ancillary and post-trial care	30	Provisions, if any, for ancillary and post-trial care, and for compensation to those who suffer harm from trial participation	N/A

For peer review only - http://bmjopen.bmj.com/site/about/guidelines.xhtml

Dissemination policy	31a	Plans for investigators and sponsor to communicate trial results to participants, healthcare professionals, the public, and other relevant groups (eg, via publication, reporting in results databases, or other data sharing arrangements), including any publication restrictions	16
	31b	Authorship eligibility guidelines and any intended use of professional writers	16
	31c	Plans, if any, for granting public access to the full protocol, participant-level dataset, and statistical code	17
**Appendices**			
Informed consent materials	32	Model consent form and other related documentation given to participants and authorised surrogates	N/A
Biological specimens	33	Plans for collection, laboratory evaluation, and storage of biological specimens for genetic or molecular analysis in the current trial and for future use in ancillary studies, if applicable	N/A

*It is strongly recommended that this checklist be read in conjunction with the SPIRIT 2013 Explanation & Elaboration for important clarification on the items. Amendments to the protocol should be tracked and dated. The SPIRIT checklist is copyrighted by the SPIRIT Group under the Creative Commons "Attribution-NonCommercial-NoDerivs 3.0 Unported" license.

For peer review only
